# A Bead‐Based Quantum Dot Immunoassay Integrated with Multi‐Module Microfluidics Enables Real‐Time Multiplexed Detection of Blood Insulin and Glucagon

**DOI:** 10.1002/advs.202412185

**Published:** 2025-04-25

**Authors:** Hesam Abouali, Sanjana Srikant, Md Fahim Al Fattah, Nicole G. Barra, Darryl Chan, Dayan Ban, Jonathan D. Schertzer, Mahla Poudineh

**Affiliations:** ^1^ Department of Electrical and Computer Engineering University of Waterloo Waterloo ON N2L 3G1 Canada; ^2^ Waterloo Institute for Nanotechnology University of Waterloo Waterloo ON N2L 3G1 Canada; ^3^ Department of Biochemistry and Biomedical Sciences McMaster University Hamilton ON L8S 4L8 Canada; ^4^ Farncombe Family Digestive Health Research Institute McMaster University Hamilton ON L8S 4L8 Canada; ^5^ Centre for Metabolism Obesity and Diabetes Research McMaster University Hamilton ON L8S 4L8 Canada

**Keywords:** diabetes hormone monitoring, glucagon, insulin, multiplexed detection, quantum dot

## Abstract

Analyzing diabetes‐related hormones such as insulin and glucagon using conventional enzyme‐linked immunosorbent assay (ELISA) has been the gold standard. However, ELISAs have a long sample processing time, including blood serum/plasma preparation which restricts the number of measurement time points, making it difficult to accurately track hormone dynamics in relation to each other and to blood glucose levels. Here, a multi‐module microfluidic platform, named quantum dot integrated real‐time (QIRT)‐ELISA system, that uses a bead‐based quantum dot‐mediated immunoassay (BQI) to continuously monitor insulin and glucagon in whole blood samples and in a multiplexed setting is demonstrated. The use of BQI simplifies the technical aspect of the system, removes the need for bulky and expensive equipment, and most importantly enhances the sensitivity, specificity, and temporal resolution. Validation experiments measuring levels of insulin and glucagon in rats during glucose tolerance tests demonstrate the accuracy of QIRT‐ELISA for simultaneously measuring endogenous hormones within the physiological range during an oral glucose load.

## Graphical abstract

The quantum dot integrated real‐time ELISA or QIRT‐ELISA system integrates a bead‐based quantum dot‐mediated immunoassay (BQI) with a modular microfluidic system to continuously monitor insulin and glucagon in whole blood samples and in a multiplexed setting. The use of BQI simplifies the technical aspect of the system, removes the need for bulky and expensive equipment, and most importantly enhances the sensitivity, specificity, and temporal resolution.

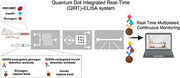

## 1 Introduction

Insulin therapy,^[^ ^1^ ^]^ coupled with advances in continuous glucose monitoring and insulin delivery technologies, has revolutionized the treatment of diabetes, and improved quality of life of patients with diabetes.^[^ ^2^ ^]^ Continuous glucose monitoring allowed dynamic, highly time‐resolved measurement of blood glucose; however, these measures do not quantify the hormones that stabilize it like glucagon and insulin. Glucagon prevents hypoglycemia while insulin promotes glucose uptake and usage into cells. Glucagon is the main hormone that counterbalances insulin action.^[^ ^3^, ^4^ ^]^ The dynamics of glucose, insulin, and glucagon in relation to one another in diabetes are not fully understood.^[^ ^5^, ^6^, ^7^ ^]^ It is important to achieve dynamic measurement of these hormones, too, because people with diabetes still experience adverse outcomes from incorrect insulin dosing due to heterogeneous insulin dynamics between individuals and even variability of insulin responses within the same person under different stressors.^[^ ^2^, ^8^ ^]^ Continuous and simultaneous measurements of insulin and glucagon would uncover how these hormones fluctuate postprandially in the progression of type 2 diabetes. The time‐course of changes in blood glucose after delivery of a glucose load is well‐established and can be rapidly and continuously measured. Currently, there is limited knowledge about the dynamic changes in blood insulin and glucagon responses, especially during a dynamic test like a glucose tolerance test (GTT). The dynamic changes between insulin and glucagon postprandially and/or after a GTT are not well‐established in a continuous and simultaneous method such as the one proposed here. Pulsatility of insulin secretion in vivo is one such dynamic change we expect to accurately detect using our detection system.

**Figure 1:**
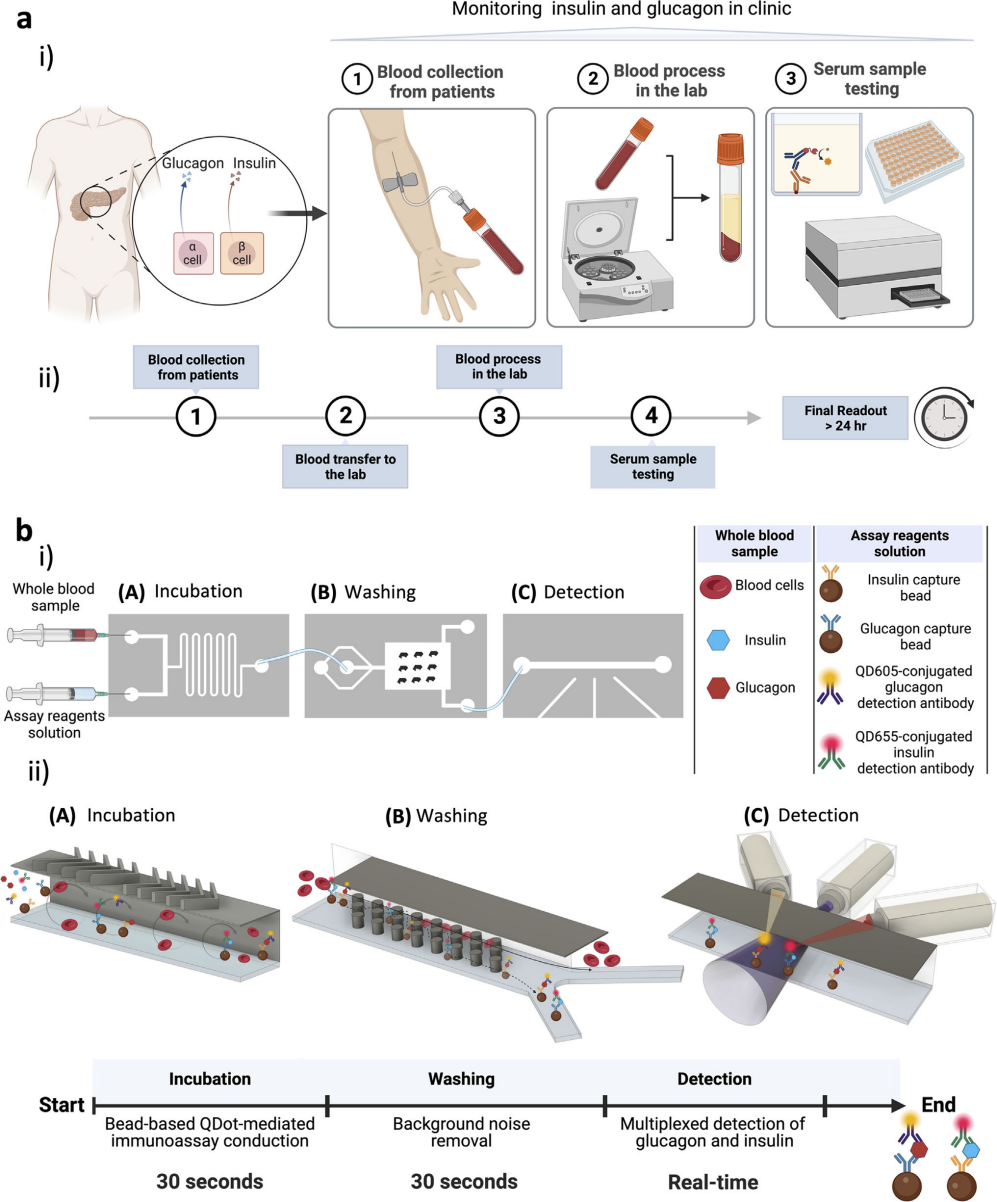
QIRT‐ELISA system for insulin and glucagon detection. a) i) The process of clinical monitoring of insulin and glucagon from whole blood samples. ii) The prolonged timeline required for this process needs more than 24 h. b) i) A schematic of the QIRT‐ELISA for real‐time, continuous measurements of insulin and glucagon. ii) 3D representation of each microfluidic module integrated into the QIRT‐ELISA system and their corresponding process‐time. Created with BioRender.com.

Continuous glucose monitoring devices that work on enzymatic detection have made frequent glucose measurement feasible, but such devices do not exist for hormones like insulin and glucagon because there is no method to track these hormones enzymatically. These analytes are in picomolar (pm) concentrations in the blood and are routinely measured by enzyme‐linked immunosorbent assay (ELISA) or techniques such as liquid chromatography coupled to tandem mass spectrometry (LC‐MS/MS).^[^ ^6^, ^7^ ^]^ Although conventional ELISA is a widely adopted method with ubiquitous applications, its utilization for measurement of hormone dynamics is limited.^[^ ^9^ ^]^ Most importantly, the ELISA protocol for glucagon involves overnight incubation and detection steps, while the insulin protocol requires a minimum of 6 h, thereby constraining the capacity to obtain measurements at only a few specific time points (as shown in **Figure** **1**ai,ii). ELISA also typically requires preparation of blood plasma or serum. These are serious limitations when it comes to determining the dynamics of insulin and glucagon in the blood for both preclinical and clinical applications.

To develop the next‐generation ELISA and reduce the amount of required reagents and completion time, ELISA was transferred into microfluidic‐based microplates.^[^ ^10^ ^]^ Bead‐based ELISA such as Luminex xMAP technology and Mercodia MBeads assay also offer scalability compared to the conventional ELISA and have increased sensitivity.^[^ ^11^, ^12^ ^]^ However, these bead‐based methods still need the preparation of plasma/serum, require the time‐consuming washing steps, and the necessary 2‐h incubation period to complete the assay, limiting their suitability for continuous measurements. In a recent work, a microfluidic platform has been developed to measure extracellular Ca^2+^ and hormones via a light‐induced fluorescence (LIF) detection setup and an ELISA assay, respectively. This platform traps a biological subject (e.g., a tissue), and measures the Ca^2+^ levels with high temporal resolution by LIF. Moreover, the secreted insulin from the subject is collected from the device and is measured using a conventional ELISA kit. By doing so, the levels of Ca^2+^ and hormone (i.e., insulin) could be studied under different conditions. The method used in this work has limitations, as the ELISA is performed off‐chip and requires a longer incubation time. Recently, an integrated microfluidic platform, called real‐time ELISA or RT‐ELISA, which benefits from a bead‐based sandwich ELISA, has been reported to continuously monitor exogenous blood insulin and glucose levels in rats.^[^ ^2^ ^]^ Although RT‐ELISA offers pm sensitivity, it relies on a bulky sCMOS camera to detect the bead‐target complex, taking up space and hindering its translation into clinical or real‐world applications. In addition, although this platform has introduced the concept of real‐time continuous ELISA measurements for the first time, the assay presents difficulties in scaling up its multiplexing capabilities. The RT‐ELISA necessitates a specific imaging setup with two lasers for two markers, making it more complex to extend the platform for additional targets, requiring a more sophisticated imaging setup and the inclusion of extra lasers for illumination.

Here, we adapt the previously developed RT‐ELISA to enable continuous, simultaneous measurement of physiologically relevant concentrations (from low pm to high pm) of endogenous insulin and glucagon in whole blood (Figure 1bi), overcoming the above mentioned limitations. A bead‐based quantum dot (QDot)‐mediated immunoassay (BQI) was developed to measure insulin and glucagon concentrations directly from whole blood continuously and simultaneously, thereby resolving limitations in conventional ELISA and bead‐based ELISAs reported in the RT‐ELISA platform.^[^ ^8^ ^]^

QDots, in contrast to traditional organic fluorescence dyes, offer advantages such as narrow emission spectra, minimal photobleaching, and increased brightness.^[^ ^13^, ^14^, ^15^, ^16^ ^]^ For our purposes, however, the most important advantage is their universal excitation wavelength.^[^ ^16^, ^17^, ^18^, ^19^ ^]^ This enables simultaneous detection of multiple targets by utilizing different QDots, all excited by a single light source (ultraviolet or blue‐light) and collecting their discrete emitted signals. Second, the on‐chip incubation decreases the completion time of the assay significantly, which is in the order of minutes, and makes real‐time measurements possible without the need to prepare serum or plasma (Figure 1bii). Third, there is no need for manual repetitive washing steps, minimizing the error in sample handling and the assay time (Figure 1bii). Finally, the inclusion of the final detection module removes the need for a bulky fluorescence microscope or sCMOS cameras (Figure 1bii). We validated the QDot integrated RT‐ELISA (QIRT‐ELISA) system for the real‐time measurement of endogenous levels of glucagon and insulin in rats during an oral glucose tolerance test. Importantly, measurements collected from QIRT‐ELISA align with those obtained from conventional ELISA, while unlike conventional ELISA, our system has the potential to assess multiplexed hormone dynamics.

## 2 Results and Discussion

### 2.1 Bead‐Based Quantum Dot‐Mediated Immunoassay Sensing Mechanism

**Figure 2:**
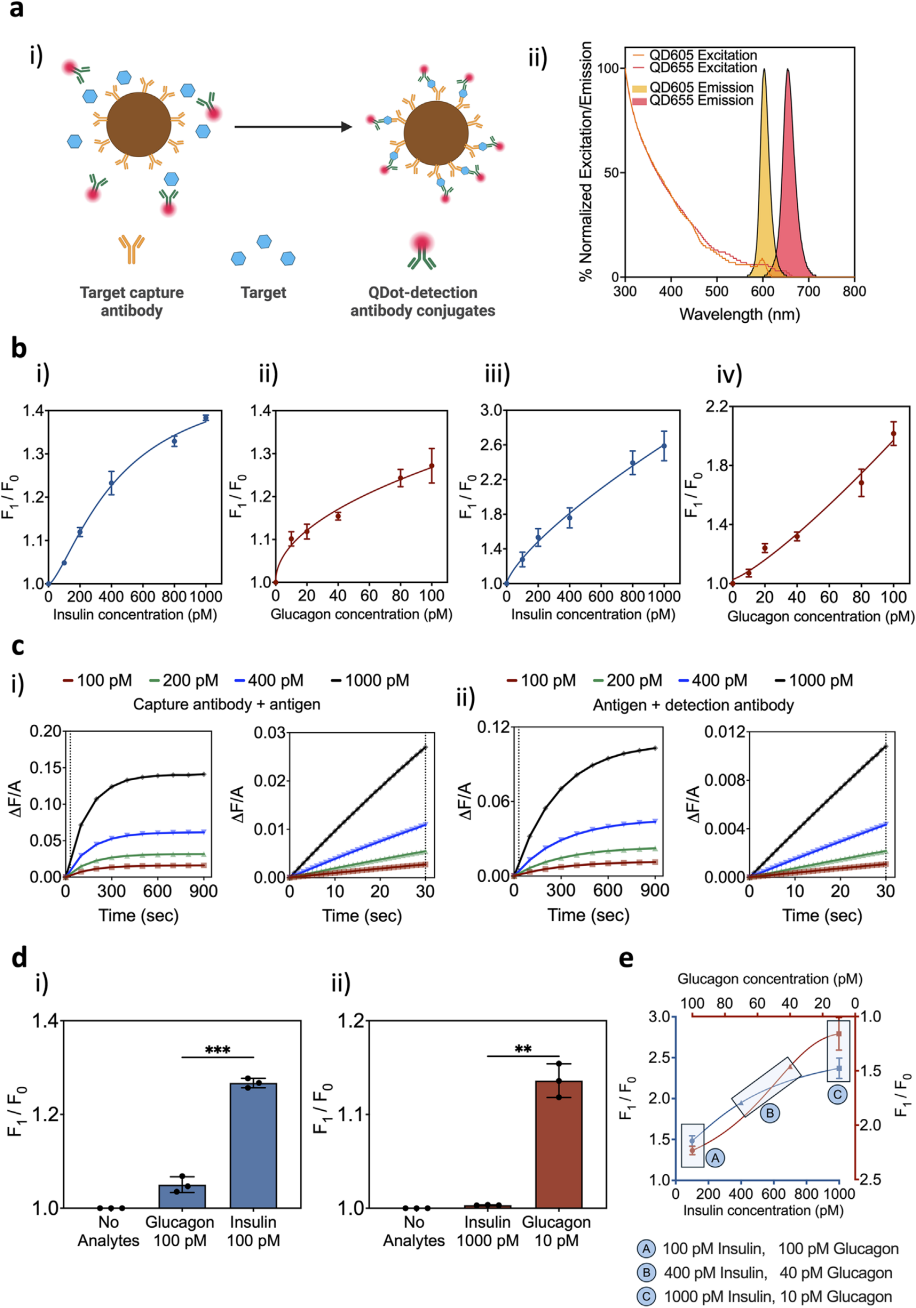
Validation of bead‐based quantum dot‐mediated immunoassay (BQI) sensing mechanism. a) i) Assay schematic employing beads functionalized with capture antibodies along with target analytes, and detection antibodies tagged with QDots to form a sandwich immunoassay. ii) Excitation and emission spectra for QDots used in BQIs and QIRT‐ELISA platform. QDot 655 and QDot 605 were used to detect insulin and glucagon, respectively. The excitation spectrum for both QDots is in the 405 nm range. b) Benchtop validation of insulin–BQI (i) and glucagon–BQI (ii) in whole blood. Insulin concentrations ranged from 100 × 10^−12^ to 1000 × 10^−12^ m, while glucagon ranged from 10 × 10^−12^ to 100 × 10^−12^ m, matching their physiological concentrations. Calibration curves for insulin–BQI (iii) and glucagon–BQI (iv) in whole blood incorporating the micromixing module for assay incubation. c) The kinetics models of the fluorescence signal intensity versus time over a time duration of 900 s for different concentrations of insulin as the model target antigen. (i) The capture antibody and antigen‐binding model results. (ii) The antigen and detection antibody‐binding model results. In panels (i) and (ii), Δ*F* = *F*(*t*) − *F*_background_, where *F*(*t*) is the fluorescence signal from the sample and *F*_background_ is the background fluorescence signal and *A* is a proportionality constant. d) Assessing the selectivity of the immunoassay of insulin (i) and glucagon (ii), the highest concentration of the nontarget analyte was assessed compared to the lowest concentration of the specific target. e) Multiplexed measurement of insulin and glucagon. Whole blood samples were spiked with insulin and glucagon concentrations that mimic their physiological trends—the lowest concentration of insulin (100 × 10^−12^ m) and the highest of glucagon (100 × 10^−12^ m) were spiked together, pair A, followed by medium‐level concentrations of both targets (insulin = 400 × 10^−12^ m and glucagon = 40 × 10^−12^ m), pair B, and finally the highest concentration of insulin (1000 × 10^−12^ m) and the lowest concentration of glucagon (10 × 10^−12^ m), pair C. *F*_0_ is the fluorescent signal for the lowest concentration (0 pm), and *F*_1_ is the fluorescent signal for each sample collected from flow cytometry. The data shows the mean ± standard deviation (SD) of three replicates. The comparisons between groups are done with unpaired t‐test with Welch's correction. *p*‐values: 0.001–0.01 (**), 0.0001–0.001 (***). Created with BioRender.com.

Similar to the original RT‐ELISA,^[^ ^2^ ^]^ bead‐based immunoassays were employed for target protein detection (**Figure** **2**ai). The magnetic beads were functionalized by anti‐rat antibodies specific to insulin or glucagon while the detection was achieved using the detection antibodies conjugated to the specific QDots as the fluorescence probe. QDot 655 and QDot 605 were used for the detection of insulin and glucagon, respectively. The excitation spectrum for both QDots is in the ultraviolet or blue‐light (405 nm) range, allowing for their multiplexed detection using a single laser excitation (Figure 2aii). Since there is no downstream analysis of captured hormones, and they were not released after their capture from the beads for downstream analysis, the effect of the capture magnetic beads on the structure of the captured hormones were not investigated.

First, we validated individual insulin‐BQI and glucagon‐BQI in buffer (Figure S1, Supporting Information) and whole blood (Figure 2b). Briefly, beads functionalized with capture antibodies and detection antibodies conjugated with QDots were incubated with their respective targets for 90 min, and after washing they were imaged using flow cytometry (Figure 2bi,ii).

Next, we studied the formation of the insulin‐BQI and glucagon‐BQI within the micromixer module of QIRT‐ELISA which mimics the incubation step (Figure 2biii,iv). The micromixer module, reported in the original RT‐ELISA,^[^ ^2^ ^]^ is a serpentine channel with embedded herringbone structures that enable chaotic and rapid mixing. The design and the details about the micromixer are available in Figure S2 (Supporting Information). The buffer or blood samples spiked with different concentrations of target analytes were injected from one inlet while the functionalized beads and detection antibodies tagged with QDots were injected from another inlet of the device at a previously optimized flow rate of 15 µL min^−2^. Upon mixing for ≈30 s, the bead–target complexes were collected from the device outlet and imaged using flow cytometry (Figure 2biii,iv).

The micromixer module outperforms the conventional assay in terms of incubation time, requiring ≈30 s, as opposed to the 6 or 36 h needed for the conventional insulin and glucagon ELISAs, respectively. This short and rapid operational incubation time is sufficient for BQI conduction which results in distinctive profiles for different concentrations (Figure 2ci,ii).

We conducted a systematic kinetics study within the mixer module. Briefly, we investigated the theoretical basis of the binding kinetics in each step of the sandwich assay. We followed the rate law for a protein–ligand reversible reaction^[^ ^20^ ^]^ and the assumptions for the enhancement in mixing by chaotic advection caused by the geometry of the micromixer which results in a substantial increase in diffusion‐limited association rates of protein‐protein complexes.^[^ ^21^, ^22^, ^23^ ^]^ The details about the kinetics model of the assay and its associated parameters can be found in Table S1 (Supporting Information). We found that the required incubation time to reach equilibrium is longer than 30 s of incubation within the mixer device (Figure 2ci). Nevertheless, this non‐equilibrium operational incubation time does not change the effectiveness of the QIRT‐ELISA system. Reaching equilibrium is not a prerequisite for accurately quantifying analyte concentrations, and many commonly used assays, such as digital ELISA^[^ ^20^, ^24^, ^25^ ^]^ function effectively under nonequilibrium conditions. The key requirement is that the output signal of our device consistently remains proportional to the analyte concentration.

We next studied the selectivity of the insulin‐BQI and glucagon‐BQI (Figure 2di,ii). When insulin (glucagon) beads and detection antibodies were incubated with the highest concentration of glucagon (insulin) in our system, the signal observed was insignificant compared to the one observed from the lowest concentration of insulin (glucagon).

The specificity of the glucagon BQI was examined against GLP‐1 in rat whole blood samples as shown in Figure S3 (Supporting Information). The results confirm that there is no cross‐reactivity between glucagon and GLP‐1 in whole blood samples, in agreement with the reported data from the manufacturer.^[^ ^26^ ^]^ We tested the stability of the quantum dots used in the insulin BQI over a two‐week period to evaluate their reliability as a fluorescence tag for detection. The results shown in Figure S4 (Supporting Information) indicate that the quantum dots used in the BQI for insulin detection remain stable for two weeks, demonstrating the long‐term stability of the BQI for insulin detection. Regarding glucagon BQI, due to the instability of antibodies at room temperature, the BQI was freshly prepared before each experiment (see the Experimental Section).

Multiplexed detection of insulin and glucagon is important because these hormones counterbalance blood glucose levels.^[^ ^5^, ^27^, ^28^ ^]^ We thus examined the micromixer module capability for simultaneous detection of insulin and glucagon using whole blood spiked with these hormones at different concentration pairs (Figure 2e). Upon target capture within the micromixer module, insulin–BQI and glucagon–BQI complexes were collected and analyzed using the flow cytometry. The collected measurements confirm that multiplexed detection of insulin and glucagon is achievable using the micromixer module.

### 2.2 Validation of Individual QIRT‐ELISA Modules

**Figure 3:**
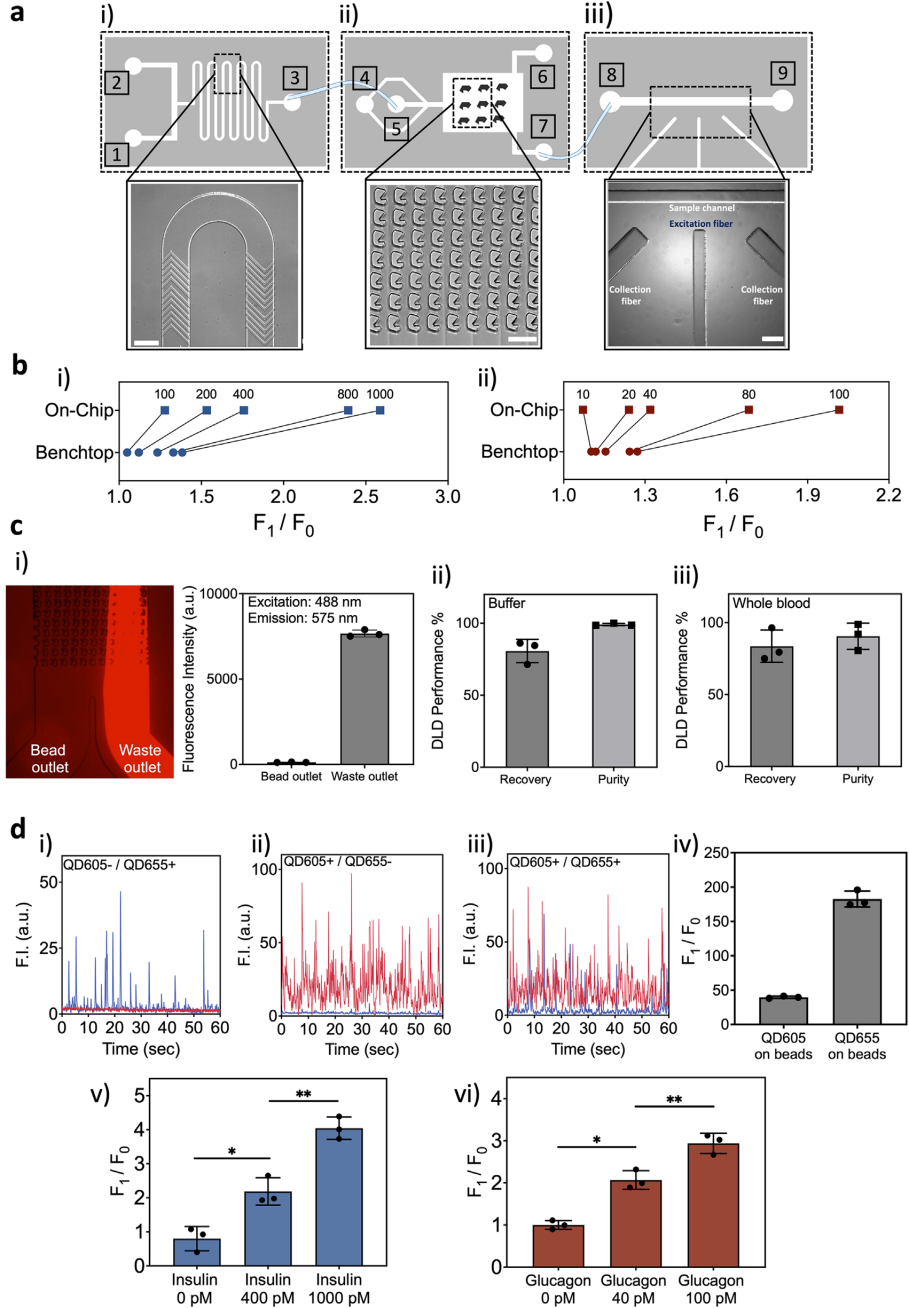
Modular device validation. a) The schematics of QIRT‐ELIA's modules are shown, with the respective microscopic images of their important features. (i) The micromixer with the herringbone structures featured on a serpentine microchannel; microscopic picture of the fabricated micromixer with the herringbone structure that generates the chaotic mixing within the microchannel. The sample enters the device from one inlet (inlet #1) and the reagents enter from the other inlet (inlet #2), and flow toward the outlet (outlet #3). Scale bar 250 µm (left). (ii) The DLD device and the pillars within it, which makes the separation of beads from the rest of the sample components possible. The formed BQI together with other unbound reagents and RBCs/WBCs enter the DLD device from the sample inlet (inlet #4), and the sheath flow helps with focusing the DLD sample inflow (inlet #5). The unwanted reagents and RBCs/WBCs follow the waste outlet streamline (outlet #6) and the BQI beads follow the bead outlet (outlet #7) which is attached to the inlet of the detection module. Scale bar 100 µm. (iii) The detection module which integrates laser fibers to distinctively collect data from the BQI samples entering from (inlet #8) and exiting from (outlet #9). The image of the detection device and the optical fiber grooves layout facing the microchannel are shown under the microscope. Scale bar 500 µm. b) Comparison of the signal ratio for different concentrations of insulin (i) and glucagon (ii). *F*_0_ is the fluorescence signal measured by flow cytometry at the lowest concentration (0 pm), while *F*_1_ corresponds to the fluorescence signal for each sample. Measurements were collected from Figure 2c (i) through (iv). c) DLD module characterization: i) The DLD device was characterized for its capability to wash free‐floating reagents which are not used in the BQI formation within the device. The collected solutions from bead and waste outlets were analyzed for the presence of PE dye, and its depletion was measured using a plate reader at its excitation and emission peaks (488 and 575 nm, respectively). The data show the mean ± SD of three replicates. The DLD device was further analyzed for its performance with two different types of samples (buffer (ii) and whole blood (iii)). The data show the mean ± SD of three replicates. d) Detection module characterization: The fluorescence signal read‐out (F.I. [a.u.]) from the detection device for QDot 605 beads (i), QDot 655 beads (ii), and a mixture of both QDot beads (iii). (iv) The area under the peak of signals (*F*_1_) for the QDot bead samples, measured by the detection system was normalized to the area under the peak of signals of bare beads (*F*_0_) as negative controls. The data shows the mean ± SD of three replicates. The area under the peak of signals for benchtop prepared insulin‐BQI (v) and glucagon‐BQI (vi) spiked in whole blood samples and analyzed with the detection module (mean ± SD of three replicates). *F*_0_ is the area under the peak of fluorescence signals for the lowest concentration (0 pm), and *F*_1_ is the area under the peak of fluorescence signals for each sample. The comparisons between groups are done with unpaired *t*‐test with Welch's correction. *p*‐values: 0.01–0.05 (*), 0.001–0.01 (**), 0.0001–0.001 (***).

The QIRT‐ELISA device consists of three modules to successfully measure the signals from insulin and glucagon continuously and in real time (**Figure** **3**a). All modules were fabricated following the standard polydimethylsiloxane (PDMS) based fabrication method. First, the micromixer or incubation device conducts the BQIs for both analytes on‐chip (Figure 3a module i); the second compartment, a washing module, helps purify the bead‐target complex from red blood cells (RBCs) and white blood cells (WBCs) and any unconjugated detection antibodies (Figure 3a module ii); finally, a new detection module was incorporated, utilizing a microfluidic channel in conjunction with optoelectronic components to measure the fluorescence signals from the BQIs (Figure 3a module iii). Incorporating QDots and implementing optofluidics for measuring fluorescence signals led to significant improvement in device performance. This was particularly notable in terms of increasing multiplexed capability and reducing the size of the device. All modules were evaluated for their modular performance before their final integration.

The micromixer module, with the embedded herringbone structures, generates transverse flow patterns that can introduce chaotic mixing in the low Reynolds numbers in the microchannels,^[^ ^29^, ^30^, ^31^ ^]^ (Figure 3ai and Figure S2, Supporting Information). By transferring the bead‐based sandwich ELISA into the micromixer, not only did the incubation time decrease significantly compared to a conventional ELISA (from hours to minutes), but also the range of the signal ratios for different concentrations significantly improve, making the differentiation between two consecutive concentrations more feasible (Figure 3bi,ii). A signal ratio range of 1.27–2.58 and 1.07–2.01 was achieved for insulin and glucagon using the micromixer device, respectively, while the benchtop assay led to 1.04–1.38 and 1.10–1.27 ranges for insulin and glucagon, respectively.

The second module of QIRT‐ELISA is a deterministic lateral displacement (DLD)^[^ ^32^ ^]^ device that washes undesired components from the formed BQIs (Figure 3aii, and Figure S5, Supporting Information). This device was designed with a critical diameter (Dc) of 14 µm and was previously optimized to efficiently separate the microbeads with a diameter of 15 µm or more.^[^ ^2^ ^]^ The DLD device has a sample inlet that is connected to the outlet of the micromixer, a sheath buffer inlet, and two outlets, bead outlet and waste outlet (Figure 3aii). Here, we validated the DLD device performance and assessed two criteria: first, the outlet purity, i.e., isolating microbeads from the whole blood components and unbound reagents, and second, the bead recovery which characterizes the number of microbeads recovered from the injected ones. First, we evaluated these two criteria in a buffer solution. The microbeads were suspended in a buffer that contained free‐floating phycoerythrin (PE)‐conjugated detection antibody. This conjugate mimicked the QDot‐conjugated detection antibody (PE dye was used for visualization purposes under a fluorescence microscope). This solution was injected through the sample inlet of the DLD device, and the outlets were collected and analyzed using a flow cytometry device for the number of microbeads recovered from the bead outlet (bead recovery calculation), and a plate reader for the concentration of PE‐detection antibody in both outlets (outlet purity calculation) (Figure 3ci,ii and Figures S6 and S7, Video S1, Supporting Information). Second, the DLD device was evaluated for these two criteria while working with rat whole blood. The number of recovered beads was counted for bead recovery calculation. Also, the number of blood cells in both bead and waste outlets was analyzed for the outlet purity calculation. Results (Figure 3ciii, and Figure S8, Supporting Information) suggest that the DLD device recovers microbeads efficiently without impurities while working with whole blood samples.

Upon passing the micromixer and washing modules, the insulin‐/glucagon‐BQIs are directed to the new detection module, where excitation and collection optical fibers were coupled within a microchannel (Figure 3aiii and Figure S9, Supporting Information). In the QIRT‐ELISA system, a 405 nm laser excites BQI conjugates. The complete optoelectronic setup^[^ ^33^ ^]^ is shown in Figure S10 (Supporting Information). The emitted fluorescence signals from QDot 655 (insulin) and QDot 605 (glucagon) were collected using two collection fibers. For initial characterization, the whole blood samples were spiked with microbeads functionalized with QDots and injected into the device. The results (Figure 3di,ii) show that the signals from both QDot beads can be detected specifically and with negligible noises for the other QDot. Another sample was prepared where both QDot beads were mixed and injected into the detection module. As shown in Figure 3diii, the multiplexed signals from both QDots were successfully monitored. The signal ratio of area under the peak of these positive controls (QDot beads) compared with the bare beads was calculated as shown in Figure 3div. Since the diameter of the BQI beads is smaller than the height of the microchannel in the detection module, beads can pass through the detection channel at varying heights. This variation in height can cause fluctuations in signal magnitude, even for the same analyte concentration. To address this issue and obtain more consistent measurements, the areas under the peaks in the collected signals were integrated as shown previously.^[^ ^33^ ^]^ This peak area integration ensures a uniform signal measurement and is applied to all subsequent measurements by the QIRT‐ELISA system, denoted by *F*_1_ or *F*_0_ values.

To further characterize the device, benchtop insulin–BQI, and glucagon–BQI samples at different concentrations were prepared and analyzed for their fluorescence signals within the detection module. The signals for each sample were considered positive signals if the recorded signal's peak amplitude exceeded 10% of the maximum peak amplitude. Any signal below this threshold was considered as noise. The area under the peak of signals for the insulin and glucagon samples was calculated and the signal ratio is shown in Figure 3dv,vi.

### 2.3 Validation of QIRT‐ELISA for Continuous, Multiplexed Measurement of Insulin and Glucagon in Hormone‐Spiked Blood Samples

**Figure 4:**
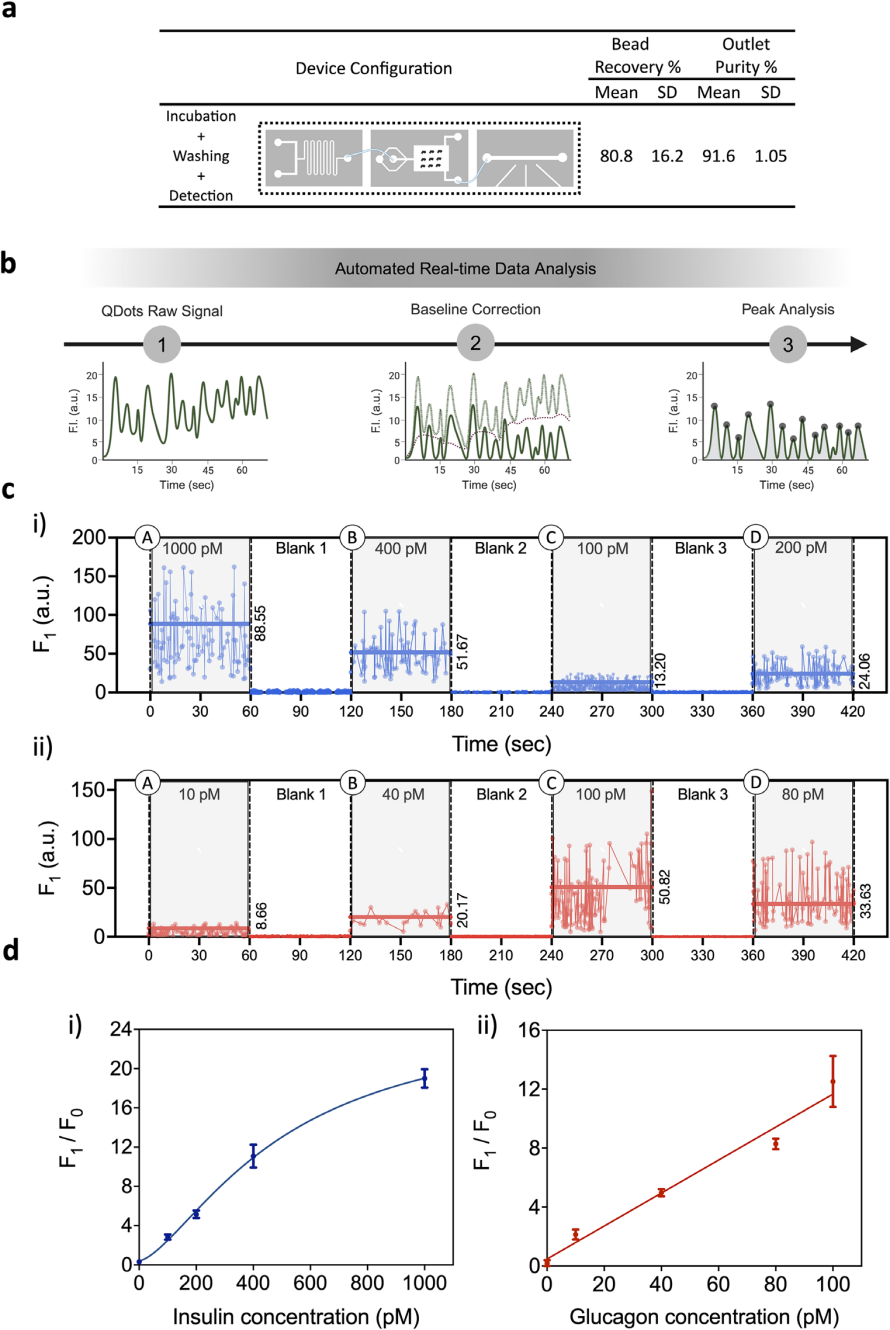
Continuous, multiplexed in vitro measurement of insulin and glucagon in whole blood. a) The results for the bead recovery and outlet purity of the integrated QIRT‐ELISA devices. b) The procedure used in the developed app to automate the analysis of collected data. First, the raw data was loaded into the app. In the next step, the baselines of the signals were adjusted to make the peak integration possible. Finally, the app determines the peaks in the signals, calculates the average area under the peaks, and outputs the concentration to build calibration curves for each target. c) Area under the peak measurements of the collected fluorescence signals for different concentrations of insulin and glucagon spiked in whole blood samples. Changes in fluorescence intensity were shown over time as different sample pairs were changed in‐line. i) Insulin continuous measurements over time. ii) Glucagon continuous measurements over time. One scan (with a duration of 60 s) is shown for each sample. d) Derived insulin (i) and glucagon (ii) calibration curves from the continuous, multiplexed measurements. The data show the mean ± SD of three scans for each sample with three replicates. *F*_0_ is the average area under the peaks for the lowest concentration (0 pm), and *F*_1_ is the average area under the peaks for each sample with a higher concentration. Created with BioRender.com.

Next, all the microfluidic modules were integrated to generate the QIRT‐ELISA system. This integration was done using silicon tubing to connect the modules together (as shown in Figure S11, Supporting Information). First, the performance of the integrated device was also characterized for its bead recovery efficiency and outlet purity. The results suggest that an average of 80% of beads are recovered from the device and there is a significant depletion of RBCs/WBCs with a purity of more than 90% (**Figure** **4**a).

Having validated the performance of the QIRT‐ELISA system for both bead recovery and purity (Figure 4a) and having demonstrated that insulin and glucagon can be measured simultaneously in the micromixer module (Figure 2e), we moved forward and examined the performance of the integrated QIRT‐ELISA for insulin and glucagon detection in whole blood samples. First, we validated the performance of the QIRT‐ELISA for detection of both analytes individually. For this purpose, whole blood samples were spiked with different concentrations of either insulin (0, 400, 1000 × 10^−12^ m) or glucagon (0, 40, 100 × 10^−12^ m). Each sample was injected into the device, and the signals were scanned for a duration of 60 s multiple times for each sample. A 30‐s time frame of one optical scan for different samples is shown in Figure S12 (Supporting Information). For 0 pm insulin, the fluorescence signal range was 3–10 a.u., for 400 × 10^−12^ m insulin, a range of 10–30 a.u. was achieved, and for the highest tested concentration of insulin, 1000 × 10^−12^ m, the signal range was 30–60 a.u. (Figure S12 i–iii). For the 0 p glucagon sample, the fluorescence signal magnitudes are in the range of 10–20 a.u., for the spiked sample of 40 × 10^−12^ m, the fluorescence signal varies in the range of 20–35 a.u., and a range of 30–120 a.u. was achieved for the 100 × 10^−12^ m sample (Figure S12iv–vi, Supporting Information).

Next, the QIRT‐ELISA platform was challenged to perform continuous and multiplexed measurements of varying insulin and glucagon concentrations. To achieve this, whole blood samples spiked with both insulin and glucagon at different concentrations were injected into the system, consequently, such that the platform measured simultaneous signals from both targets for a duration of 1 min. Whole blood samples were spiked with physiological concentrations of insulin and glucagon in blood. This included 1000 × 10^−12^ m insulin and 10 × 10^−12^ m glucagon (sample pair A), 400 × 10^−12^ insulin and 40 × 10^−12^ glucagon (sample pair B), 100 × 10^−12^ m insulin and 100 × 10^−12^ m glucagon (sample pair C), and finally 200 × 10^−12^ m insulin and 80 × 10^−12^ m glucagon (sample pair D). Between samples with different concentrations, blank samples were run (whole blood without any insulin or glucagon being added) to capture the change in the fluorescence readout of the system. The optical scans of each sample were analyzed using a developed app to promptly calculate the area under the peaks, facilitating real‐time measurements. Figure 4b shows the procedure implemented in the app (available on Github^[^ ^34^ ^]^). An example of this area integration is shown in Figure S13 (Supporting Information). Figure 4ci,ii shows the area under the peak of the fluorescence signal measurements from optical scans of each sample (with both targets) and their fluctuations over time as samples were switched in and out. The variation in the number of scans among different samples primarily results from the settling of beads in the syringe during measurements. A scan is considered successful if a minimum of 10 peaks, indicative of beads entering the detection channel, are observed within a 30‐s time frame. On average, 30 peaks within a 30‐s time frame were observed in the continuous measurement experiments. The area under the peak of the fluorescence signal measurements was then used to derive calibration curves for multiplexed detection of insulin and glucagon (Figure 4di,ii). The QIRT‐ELISA achieves the insulin or glucagon measurement within 60–70 s (from the moment samples are introduced into the micromixer module until the fluorescence measurements are taken using the detection module). This represents a substantial step forward from conventional ELISAs, which typically require hours to days for measurement. These findings establish the QIRT‐ELISA as a real‐time system capable of directly measuring insulin and glucagon concentrations in whole blood within clinical or preclinical environments. We calculated the limit of detection (LOD) of the integrated QIRT‐ELIS based on the standard deviation and the slope of the calibration curve and found that our device achieved an LOD of 27.4 × 10^−12^ m for insulin and 1 × 10^−12^ m for glucagon measurements in blood. The previously published, original RT‐ELISA^[^ ^2^ ^]^ had an insulin LOD of 109 × 10^−12^ m. Hence, the new QIRT‐ELISA has a higher sensitivity. This improvement is primarily attributed to the utilization of QDots as the fluorescence reporter and the implementation of highly sensitive fiber optic excitation and collection methods. The improved sensitivity now allows for the detection of fasting insulin in non‐diabetic patients (≈40 × 10^−12^ m) as well as postprandial insulin levels, thereby surpassing the performance of the original RT‐ELISA. The obtained glucagon LOD also covers the physiological range of blood glucagon in the fed and fasted states even when multiplexed with insulin measurements. The LOD for glucagon covers the expected levels of blood glucagon during euglycemia (≈5 × 10^−12^ m), hypoglycemia (up to 40 × 10^−12^ m), and possible even during hyperglycemia (<5 × 10^−12^ m).

### 2.4 In Vivo Validation of QIRT‐ELISA for Measuring Endogenous Insulin and Glucagon

After demonstrating the capability of QIRT‐ELISA to measure insulin and glucagon in spiked blood samples, we next assessed the system's efficacy in measuring their endogenous levels in vivo. The GTT is a particularly useful preclinical and clinical assessment of blood glucose control. For these experiments, to collect enough blood samples for both QIRT‐ELISA and conventional ELISA measurements, each discrete GTT experiment was conducted using three rats, with one rat assigned to each specific time point (T0, T15, and T60). In these experiments, for a single GTT experiment, three rats were fasted overnight, and then the first fasted rat was used to measure T0. The other two rats were gavaged with glucose at T0; then, the second rat was sacrificed at T15 post‐gavage, and the third rat was sacrificed at T60 post‐gavage.

**Figure 5:**
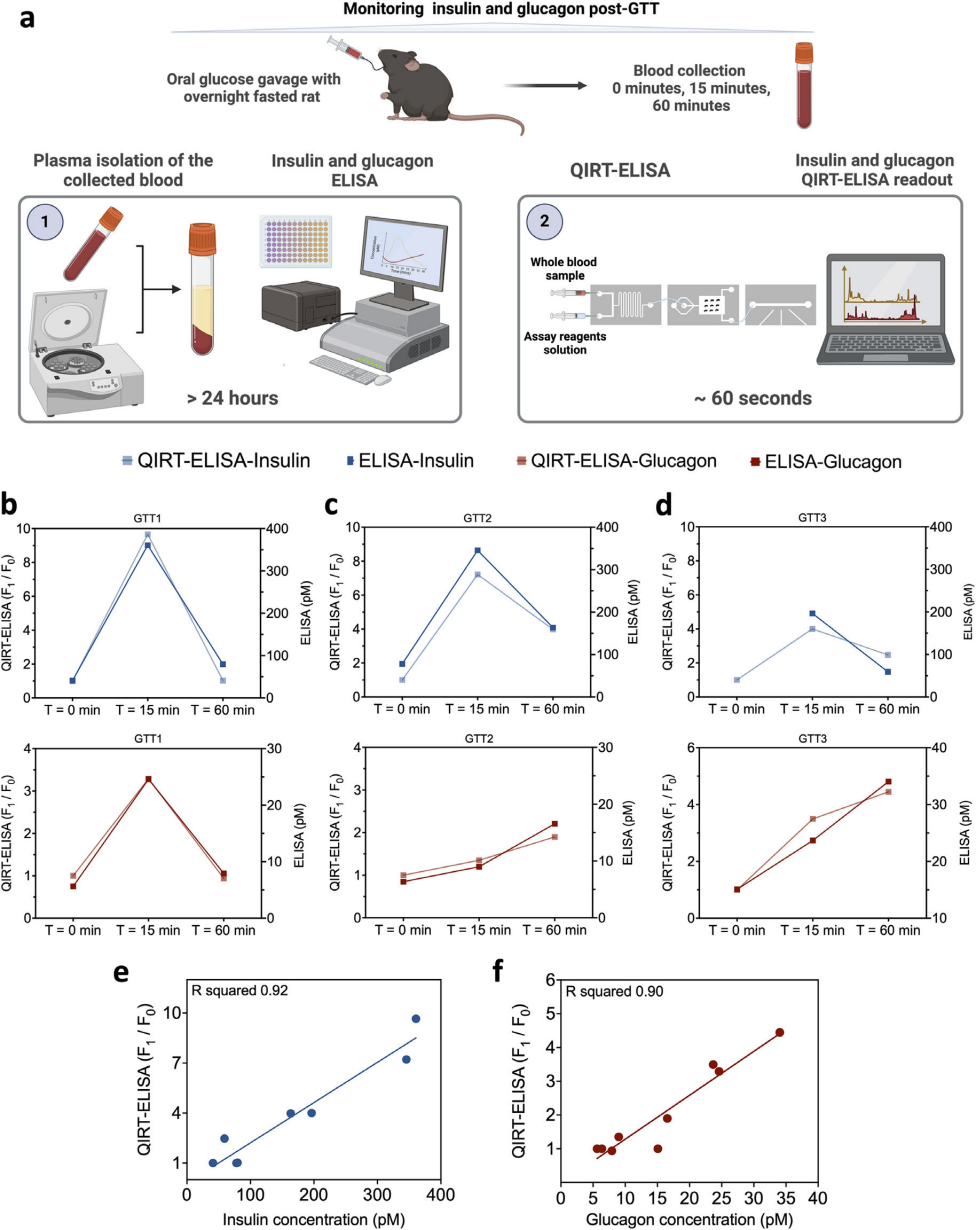
In vivo measurements of endogenous insulin and glucagon post‐glucose tolerance test (GTT). a) Schematic of GTT in rats followed by measurements of insulin and glucagon through conventional ELISA and QIRT‐ELISA in rat models. b) First GTT experiment, c) the second experiment GTT, and d) the third GTT experiment showing trends of insulin and glucagon empirical concentrations obtained by conventional ELISA along with the corresponding ratio of the average area under the peaks reading from QIRT‐ELISA across three time points following glucose administration. In (b)–(d), insulin data are shown in blue and glucagon data are shown in red. The average area under the peaks at *T* = 0 min was chosen as *F*_0_ for normalization. In (d), insulin ELISA fails to report a value for *T* = 0 min. All measurements of insulin and glucagon from blood draws at each time point were performed in a multiplexed manner with the QIRT‐ELISA platform, while the cross‐validation using ELISA measurements were performed using two separate conventional ELISA kits for insulin and glucagon. e,f) The correlation between QIRT‐ELSIA measurements and ELIA for insulin (i) and glucagon (ii) using the collected data of the three GTT experiments. Table S2 (Supporting Information) summarizes the collected measurements. Created with BioRender.com.

The collected blood samples were then tested with QIRT‐ELISA and conventional ELISA to establish trends in insulin and glucagon (as illustrated in **Figure** **5**a). Two sets of GTT were performed and the QIRT‐ELISA system was used for multiplexed hormone measurements (Figure 5b,c and Table S2, Supporting Information). Before the oral glucose load (i.e., at time zero), insulin levels were low, but insulin peaked at 15 min after glucose administration, and then decreased, returning to nearly the initial fasting levels by the 60‐min post‐glucose administration. The GTT did not significantly alter glucagon levels. The results shown in Figure 5b,c demonstrate that QIRT‐ELISA measurements closely correlated with those obtained from the current gold‐standard ELISA using blood plasma (Figure 5di,ii).

Glucagon secretion is known to be suppressed after an oral glucose load; however, the results in Figure 5 do not show this trend. We believe that the abnormal glucagon levels observed in these experiments may be due to the metabolic stress induced by isoflurane anesthesia, as the GTT experiments were conducted on anesthetized rats.^[^ ^35^, ^36^, ^37^, ^38^ ^]^ Another reason we do not see a continuous drop in glucagon from baseline throughout the GTT is likely due to how the experiment was initially conducted. A rat was sacrificed at each timepoint to collect blood and so the baseline was not quantified for each rat. It is worth noting that this part of the study aimed to cross‐validate QIRT‐ELISA with the conventional ELISA and show that there is an agreement between these two methods.

### 2.5 Continuous In Vivo Measurement of Endogenous Insulin and Glucagon in Rat Models

**Figure 6:**
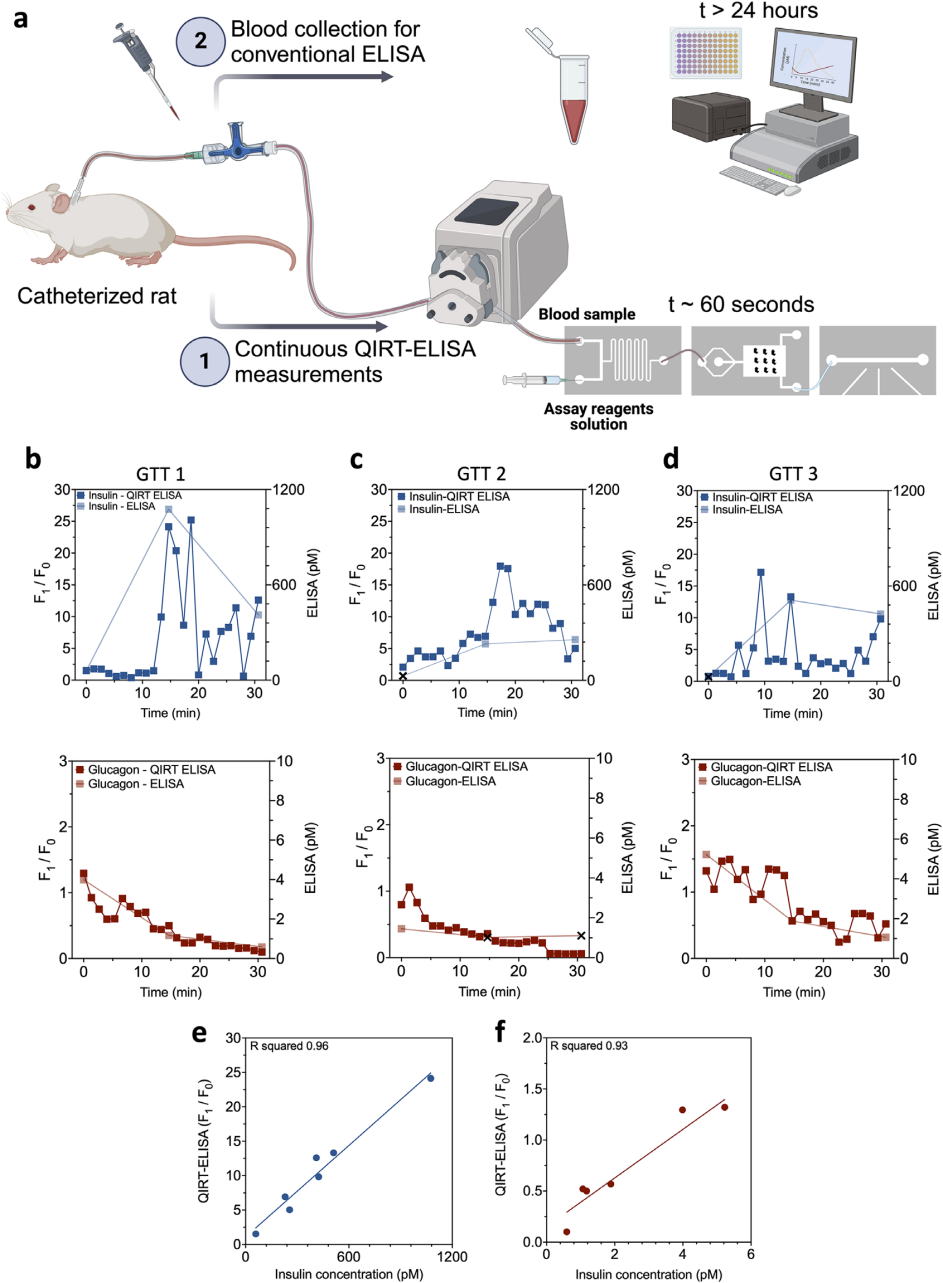
Continuous measurement of endogenous insulin and glucagon in conscious rat models. a) Schematic of the experiment process. A peristaltic pump continuously withdrew blood from the conscious rat models, and the blood was injected into the QIRT‐ELISA device in each GTT experiment. The same withdrawn blood was sampled to measure endogenous hormone production using conventional ELISAs at 0‐, 15‐, and 30‐min time points. The QIRT‐ELISA measured insulin and glucose continuously for a duration of 30 min in b) GTT experiment #1 in the first rat, c) GTT experiment #2 in the second, and d) GTT experiment #3 in the third rat. e,f) The correlation between QIRT‐ELSIA measurements and ELIA for insulin and glucagon using the collected data of the three GTT experiments. Table S3 (Supporting Information) summarizes the collected measurements. Fluorescence signals (*F*_1_/*F*_0_) values were measured and compared to the ELISA at three main time points. Each data point in the QIRT‐ELISA graphs shows the average *F*_1_ [a.u.] values for a scan with a duration of 60–80 s normalized to average fluorescence signals values prior to glucose injection (*F*_0_). The data points that the conventional ELISA fails to report a value are shown with a black cross sign. Created with BioRender.com.

The QIRT‐ELISA system was evaluated for an improved time resolution in the measurement of insulin and glucagon during an oral GTT in conscious rat model. Rats were fasted overnight and, on the day of experiment, were administered a 5 g kg^−1^ dose of glucose intraperitoneally as previously outlined in the manuscript. The rat was connected to a peristaltic pump, with blood withdrawn at a flow rate of 15 µL min^−1^ and injected into the QIRT‐ELISA device (**Figures** **6**a and S11, Supporting Information). The reagents were injected into the reagent inlet at the same time using a syringe pump. The optical system analyzed the levels of insulin and glucagon in whole blood for a duration of 30 min. The blood samples were collected from the rat at different time points to measure circulating insulin and glucagon levels using conventional ELISA. The results included in Figure 6 show that the QIRT‐ELISA system detects the levels of both targets in the blood samples with a good correlation (Figure 6e,f and Figure S14, Supporting Information) in values compared with conventional ELISA. The QIRT‐ELISA system offers enhanced time resolution compared to the conventional ELISA by measuring insulin and glucose levels in 60–80 s time intervals during the experimental time. The QIRT‐ELISA provided at least 22 measurements, while the conventional ELISA measurements were limited to three data points in a 30‐min period.

The results shown in Figure 6b–d suggest that at time point 0 min of the GTT experiment, the QIRT‐ELISA system provides higher precision in the detection of insulin while the gold‐standard conventional ELISA fails to provide an exact value at time point 0 min for GTT 2 and GTT 3 since the read‐out concentration is below the ELISA LOD (data points shown with a black cross sign). Interestingly, in both first and third GTT experiments, the biphasic insulin release previously reported during GTT was detected using QIRT‐ELISA measurements. In contrast, conventional ELISA failed to capture this due to its limited data points. These rapid real‐time concentration measurements cannot be done by conventional ELISA since the sampling volume is restricted, and the process of drawing blood frequently is also time‐consuming and labor‐intensive, stressful for the rats, and may not meet ethical criteria. The biphasic blood glucose responses are associated with increased blood levels of insulin and glucagon‐like peptide 1 (GLP‐1).^[^ ^18^ ^]^ In addition, it is known that insulin release occurs in a biphasic response in vitro and in vivo.^[^ ^19^ ^]^ The cell biology/physiology of biphasic insulin release has been extensively studied.^[^ ^20^ ^]^ However, very little is known about the in vivo dynamics of blood insulin and glucagon during a glucose load. Technical limitations have limited assessment, which may be more complex than biphasic responses. QIRT‐ELISA allows for testing how hormones dynamics correlate with different blood glucose responses in health and disease.

Moreover, in the second GTT, results show that insulin peak happens later than 15 min post‐GTT, which the conventional ELISA failed to capture since it cannot be conducted as frequently as a continuous monitoring system (Figure 6b). In this GTT experiment, a possible sample loss during the ELISA procedure may have resulted in the inability to report a glucagon concentration value at the 0‐min time point. Finally, the same experimental result demonstrates that the conventional ELISA fails again to report time points 15‐ and 30‐min post‐GTT since the concentrations are below the assay LOD (data points shown with a black cross sign) (Figure 6b).

The insulin results in Figure 6d also show the potential of the QIRT‐ELISA system in continuous monitoring since the data shows that the actual peak insulin response is happening around time point 10 min, which will not be possible to capture if we conduct the conventional ELISA with the preconceived idea that insulin peak happens at time point 15 min.

Finally, these results confirm that with the utilization of QIRT‐ELISA for in vivo continuous experiments with conscious rat models, we could observe the expected physiologically relevant trends of insulin and glucagon after GTT administration (Figure 6), whereas the results from the unconscious rat model experiments shown in Figure 5 shows that such a trend could not be seen in the rats under anesthesia.

### 2.6 Outlook

We demonstrate the first microfluidic technology capable of continuously detecting multiplexed insulin and glucagon in real‐time. The utilization of QDots as fluorescence probes in the BQI allows for the system miniaturization and enables rapid and simultaneous detection of physiological concentrations of insulin and glucagon in whole blood. The integrated QIRT‐ELISA system has been demonstrated to effectively and continuously monitor fluctuating and multiplexed concentrations of insulin and glucagon spiked into whole blood samples. Specifically, the QIRT‐ELISA achieves an LOD of 27.4 × 10^−12^ and 1 × 10^−12^ m for the detection of insulin and glucagon, respectively. The obtained LODs cover insulin and glucagon blood levels that are relevant to people with and without diabetes. Finally, the QIRT‐ELISA was used to assess the endogenous levels of insulin and glucagon in blood samples obtained from rats during the GTT. The results obtained from QIRT‐ELISA in the collected whole blood closely match the results obtained using standard ELISAs in blood plasma. Notably, QIRT‐ELISA potentially allows for the rapid detection of changes in insulin and glucagon levels, with a sample processing and detection time of approximately one minute. This significant decrease in assay time compared to other technologies along with the advantages of the BQI, makes QIRT‐ELISA a better candidate compared to recent microfluidic platforms^[^ ^2^, ^10^, ^39^, ^40^ ^]^ and represents an innovative ELISA to overcome the challenges and key shortcomings of the conventional ELISA (Table S4, Supporting Information). We next connected the QIRT‐ELISA to the conscious rats for continuous, real‐time measurements in vivo. This allowed us to study hormone dynamics in pre‐clinical rat models. The results showed that the QIRT‐ELISA system provides more useful information compared to conventional ELISA, addressing the challenges associated with this gold‐standard method.

In the future work, we will study rat models representing complex physiological conditions such as obesity, prediabetes, and diabetes, providing valuable insights into the progression of diabetes. Combining dynamic measurements of hormones like insulin and glucagon with continuous monitoring of key peptides such as C‐peptide and glucose will help bridge a critical knowledge gap. Current platforms for assessing these hormones capture only a few time points with significant delays, limiting their ability to provide highly time‐resolved data. This limitation is a major challenge in both preclinical and clinical assessments. Continuous and simultaneous measurement of insulin, glucagon, C‐peptide, and glucose with the QIRT‐ELISA system will be necessary and it will provide new fundamental knowledge of how factors beyond blood glucose are involved in the progression of diabetes and add critical information for personalized nutrition.

## 3 Experimental Section

### Materials

Chemicals and kits were purchased from Sigma Aldrich and Thermofisher, respectively, unless otherwise mentioned. Carboxylate‐functionalized magnetic 15 µm microspheres were purchased from Spherotech Inc. Matched pair antibodies against insulin and glucagon were purchased from Mercodia. Insulin antigen was obtained from Toronto Biosciences, and glucagon antigen was purchased from Prospec Bio. The ELISA kits for insulin and glucagon were purchased from Mercodia. Please see Table S5 (Supporting Information) for the list of the reagents and buffers used in this work.

### Microbead Functionalization

Carboxylate‐modified beads were based on the manufacturer's protocol for functionalizing 15 µm beads with immunoglobulins. Briefly, beads were washed on a magnetic rack and incubated for 30 min in 2‐(N‐morpholino)ethanesulfonic acid buffer (MES) buffer (C_6_H_13_NO_4_S, MW = 195.2 g mol^−1^, pH 5.2) with N‐hydroxysuccinimide (NHS) and 1‐ethyl‐3‐(3‐dimethylaminopropyl)carbodiimide (EDC) to activate carboxylate groups on the surface. Beads were then incubated with capture antibodies for 4 h in pH 7.5 MES buffer, before being quenched for 30 min in a 30 × 10^−3^ m glycine solution in BSA to terminate coupling reaction. Beads were finally washed with 1× PBS + 0.05% Tween‐20 (1× PBST) and resuspended in 1× PBS + 0.01% Tween‐20 + 0.05% BSA (1× PBSBT). Glucagon capture beads were aliquoted and stored at −20 °C and insulin capture beads were stored at 4 °C.

### Benchtop BQI

Detection antibodies against glucagon were biotinylated as per protocol outlined in the Pierce Antibody Biotinylation kit for IP, and detection antibodies against insulin were conjugated with QD655 with a SiteClick kit. Biotinylated glucagon detection antibodies were aliquoted and stored in −20 °C and Qdot conjugated insulin detection antibodies were stored in 4 °C. Prior to their use in assay experiment, glucagon detection antibodies were incubated with 50 × 10^−9^ m Streptavidin‐Qdot 605 to for a detection antibody‐Qdot complex for glucagon detection.

To complete the immunoassay complex, and validate the performance of the respective matched pair antibodies, capture beads and detection antibodies were incubated on the benchtop with increasing concentrations of target antigen for 90 min, before being washed on the mag rack with 1× PBST and analyzed by a flow cytometer (CytoFlex, Beckman Coulter). Calibration curves for each target were generated after benchtop incubations with capture beads and detection antibodies, both in 1× PBSBT buffer and in whole blood. For glucagon antibodies, the recommended short‐ and long‐term storage temperature is −20 °C. As a result, the SiteClick conjugation protocol could not be used for quantum dot conjugation. This is because the SiteClick labeling kit requires three specific overnight incubations at room temperature, which would compromise the stability and viability of the glucagon detection antibody. Therefore, to maintain the viability of the antibodies, the biotinylated detection antibody was conjugated with Streptavidin‐Qdot605 for half an hour at room temperature immediately before use in the experiment. It should also be noted that quantum dots should not be frozen—this causes formation of aggregates and loss of their functionality—thus, the best way to ensure a functional quantum dot‐mediated bead‐based assay is to maintain the antibodies at −20 °C before use and functionalize them with Streptavidin‐Qdot605 shortly before performing an experiment.

### On‐Chip BQI

Once these were established, the immunoassay was incorporated into the microfluidic micromixer module to validate the assay's performance on chip. Devices were fabricated and degassed as outlined in the device fabrication protocol. A syringe filled with assay reagents (capture beads and detection antibodies) and another filled with whole blood spiked with target antigen to a final volume of 200 µL were fitted into a syringe pump and flown through the degassed microfluidic micromixer module to perform the previously described benchtop immunoassay in minutes. A flow rate of 15 µL min^−1^ was used and the solution collected in the outlet of the micromixer module was then washed with 1× PBST before subjecting it to flow cytometry analysis (CytoFlex, Beckman‐Coulter).

### QIRT‐ELISA Device Fabrication

First, the microchannels were designed on AutoCAD and KLayout software. The masks needed for the two‐layer photolithography of the micromixer and the detection module were printed (CAD/Art Services). An SU‐8 3050 (MicroChem) layer of 45 µm was coated on a Silicon wafer (University Wafer) for the first layer, which was the main channel of the micromixer. Then the same height of SU‐8 3050 was coated on the developed first layer to pattern the herringbone structures. Both steps were fabricated using a mask aligner with a UV light (MA6, SUSS MicroTec). The process for the master mold of the washing (DLD) module followed a one‐layer fabrication protocol. The channel height for the devices (also including the micropost) was 50 µm, and it was carried out using a UV Direct Write photolithography (MLA 150, Heidelberg Instruments). The detection module was fabricated through the conventional photolithography method as well. The first layer, which was for the main channel and has a thickness of 250 µm, was done using SU‐8 2100 (MicroChem). The second layer, which included the fabrication of optical fiber grooves, followed a similar approach. The microfluidic devices were fabricated following a standard PDMS‐based method. The casted PDMS on the master molds were peeled off and bonded to glass slides (Electron Microscopy Sciences) using a plasma etcher (Tergeo Plasma Cleaner, Pie Scientific) with the following setting (15 W, 30 s, 10 sccm, air). Prior to the experiment, all the devices were conditioned and degassed with a nonionic surfactant, Pluronic F108 (Sigma Aldrich) overnight.

### Flow Cytometry for Device Characterization

The performance of the modules regarding the bead recovery and purity was evaluated using a flow cytometry device (NovoCyte, Agilent). For each integration, the number of collected beads from the bead outlet of the washing module or the detection outlet was analyzed and compared to the number of loaded microbeads (20000) at the inlet. The purity of each integration was assessed with a similar approach. The obtained FCS/SSC parameter results were gated for the population of RBCs/WBCs in the mentioned outlets, and their numbers were compared to their numbers in the waste outlet of the washing module.

### Continuous In Vitro Measurement of Insulin and Glucagon

QIRT‐ELISA devices were fabricated and degassed as outlined in the device fabrication protocol. A syringe filled with a larger volume (≈1 mL) of assay reagents (capture beads and detection antibodies) and another (sample syringe) filled with whole blood spiked with target antigens to a final volume of 200 µL were fitted into a syringe pump and flown through the degassed QIRT‐ELISA platform. Each sample syringe contained a distinct test condition. Test conditions were as follows: 1000 × 10^−12^ m insulin with 10 × 10^−12^ m glucagon, 400 × 10^−12^ m insulin with 40 × 10^−12^ m glucagon, 200 × 10^−12^ m insulin, with 80 × 10^−12^ m glucagon, and 100 × 10^−12^ m insulin with 100 × 10^−12^ m glucagon. Between each concentration, a wash sample, i.e., a whole blood sample without any spiked insulin or glucagon was processed. Sample syringes were each run until enough volume to obtain signals for 3–4 min. The sample syringe was then switched out to the next consecutive sample, while keeping the reagents syringe unchanged. A flow rate of 15 µL min^−1^ was used for injecting reagents and samples into the micromixer module, and a flow rate of 75 µL min^−1^ was used as the DLD sheath flow rate. Samples were run until sufficient peaks were obtained for both targets.

### Continuous In Vivo Measurement of Endogenous Insulin and Glucagon in Rat Models

A ten‐week‐old male Sprague Dawley rats with an indwelling catheter surgically placed in the jugular vein were purchased from Charles River. The rat was fasted overnight for ≈12 h. After weighing, the rat was subcutaneously injected with saline every hour for 4 h. Throughout the duration of the test, the rat was housed in a cage placed on a heating pad. The catheter was flushed with 100 µL/100 g BW of 100U heparin followed by 100 µL/100 g BW of saline based on the rat's body weight to maintain catheter patency. The catheter was attached to the QIRT‐ELISA device and blood was then withdrawn. After 10 min, the rat was injected with 5 g kg^−1^ of glucose intraperitoneally.

For blood collection, the catheterized rats were connected to a peristaltic pump (ISMATEC), and blood was collected continuously at a flow rate of 15 µL min^−1^ (the optimized flow rate of the micromixer) and injected into the device. The withdrawn blood was sampled at time points 0 (baseline), 10, and 30 of the experiment through a valve incorporated into the pump tubing. The reagents were injected into the device using a syringe pump, and the optoelectronic system measured the signals from both targets. Here, a single rat was used per GTT experiment. In total, three GTT experiments were conducted with three different rats.

### App for Continuous Experiment Data Analysis

To facilitate the automated and rapid data analysis for the continuous experiment, a custom app was developed. In this app, the data files from the oscilloscope were exported to the computer and imported to the app. In the first step, the app processes the data files, and extracts information for both insulin and glucagon. Next, a package (PeakUtils) that utilizes an iterative polynomial regression algorithm was used to estimate and remove the undesired baseline from the signals, making it possible to calculate the area under the peaks accurately. In the next stage, the app finds the peaks in the signals if the condition of exceeding 10% of the maximum peak amplitude is satisfied in the signal peaks and integrates the area under each peak. The algorithm uses the find_peaks package from the SciPy library to find the peaks by finding the local maxima and uses the peak_widths package to calculate the peak width, noting the height and prominence of the peak. Then, the algorithm calculates the area under the peaks (*F*_1_) following the trapezoidal rule, using the numpy.trapz package from the NumPy library. Finally, the average area under the peaks for each scan is translated into the normalized signal readout, and using the derived calibration curves, the app calculates the concentration from each scan. The developed code in the app can be found online at https://github.com/hesam‐abouali/2B‐QIRT‐ELISA and https://doi.org/10.5281/zenodo.13324474 or the app can directly be used from the Streamlit web apps repository at https://2b‐qirt‐elisa.streamlit.app.

### Optofluidic Setup

The foundation of the system (Figure S10, Supporting Information) lies in the use of QDot 605 and QDot 655 for tagging the respective biomarkers and collecting the fluorescence signal when excited with a 405 nm laser (Model: LP405‐MF300, Thorlabs) having 300 mW optical power to microfluidic channel‐bound beads. The incorporation of a 260 µm (Model: M137L02, NA: 0.22, Thorlabs) optical fiber, inserted within a groove in the microfluidic chip precisely guides the laser light, minimizing energy loss, reducing light divergence, and allowing concentrated illumination at the point of interrogation. Simultaneously, two additional 425 µm (Model: M118L02, NA: 0.39, Thorlabs) optical fibers were introduced in two other grooves at a 45° angle on the same side as the excitation fiber, facilitating the collection of emitted fluorescence signal without direct exposure to the excitation light minimizing the interference effect. Two bandpass filters, 610/10 nm (Model: FBH610‐10, Thorlabs) and 660/10 nm (Model: FBH660‐10, Thorlabs), were chosen to allow only the relevant fluorescence from quantum dots to pass through. The filtered fluorescence light was focused through a focusing lens (Model: LB1494‐A, Thorlabs) before being directed onto the detector's active area. Two Si‐pin power photodiodes (Model: 818‐UV/DB, Newport) were used to convert the fluorescence signal into an electrical signal. To overcome the inherent challenges of collecting electrical signal equivalent to the weak optical signal from the beads conjugated with quantum dots, the use of two low noise current preamplifiers (Model: SR570, Stanford Research System) significantly enhances the system's sensitivity, ensuring the reliable capture of even subtle signals. The system's output was interfaced with an oscilloscope connected to a laptop, providing a user‐friendly platform for real‐time data visualization.

### Glucose Tolerance Test

All animal procedures were approved by the Animal Research Ethics Board of McMaster University. Three male Wistar rats (Charles River) at 9–12 weeks of age were fasted overnight for 16 h per GTT experiment. The next morning, blood was drawn before (i.e., time = 0) or 15 or 60 min after oral gavage of 8 g kg^−1^ d‐glucose (Sigma). For blood collection, rats were anesthetized using isoflurane, and blood was drawn from the aorta and placed into EDTA‐coated tubes (BD). In total, three GTT experiments were conducted. In each GTT experiment, three rats were used to measure different time points. For insulin and glucagon ELISAs (Mercodia: insulin 10‐1250‐01, glucagon 10‐1281‐01), a portion of blood was centrifuged at 10000 *g* for 10 min at 4 °C to separate and collect plasma, and then subsequently stored at −80 °C until needed.

### Statistical Analysis

All the statistical analyses were conducted using GraphPad Prism 10. The statistical difference between groups in modular and integrated devices was analyzed using a two‐tailed unpaired *t*‐test with Welch's corrections. The significance of the statistical difference was calculated with 95% confidence interval (*P* < 0.05) and shown in GP style (≥0.05 (ns), 0.01–0.05 (*), 0.001–0.01 (**), 0.0001–0.001 (***), <0.0001 (****)) in graphs. Each experiment was carried out in three parallel replicates. All data are demonstrated as mean ± SD of three experimental replicates or three spectral scans. The calibration curves were calculated using the sigmoidal 4PL nonlinear regression model. The LOD is defined as the minimum target concentration that can be detected by every benchtop or on‐chip BQI and calculated by LOD  =  3.3  ×  SD / S where SD is the standard deviation and *S* is the slope of the calibration curve.

### Animal Research Ethics

All animal procedures were approved by the McMaster Animal Research Ethics Board (AREB) (approval number 23‐64), and all animal in vivo experiments were followed by these procedures and guidelines.

## Conflict of Interest

The authors declare no conflict of interest.

## Author Contributions

H.A., S.S., and F.F. contributed equally to this work. H.A. fabricated modular and integrated microfluidic devices, conducted experiments for device evaluation, and developed the code for the QIRT‐ELISA app for continuous measurements. S.S. designed and validated BQI assays and conducted experiments for device evaluation. F.F. developed the optical detection part integrating the photonic components with the microfluidics for real‐time continuous detection of multiple biomarkers. H.A., S.S., and N.B. conducted all experiments for GTT in vivo measurements. H.A., S.S., F.F., N.B., D.B., J.S., and M.P. conceived the initial concept and designed experiments. H.A., S.S., F.F., N.B., and D.C. executed experiments and analyzed the data. H.A., S.S., and M.P. wrote the manuscript. All authors edited, discussed, and approved the whole paper.

## Code Availability

The developed code in the app can be found online at https://github.com/hesam‐abouali/2B‐QIRT‐ELISA or the app can directly be used from the Streamlit web apps repository at https://2b‐qirt‐elisa.streamlit.app.

## Funding Sources

This research was supported by the Canadian Institutes of Health Research grant to Dr. Poudineh and Dr. Schertzer (202104PJT‐461445‐DOL‐CENA‐79527), and Natural Sciences and Engineering Research Council of Canada grant to Dr. Ban and Dr. Poudineh.

## Supporting information

Supporting Information

Supporting Information

## Data Availability

The data that support the findings of this study are available from the corresponding author upon reasonable request.
