# Supplementary material for: A Bead‐Based Quantum Dot Immunoassay Integrated with Multi‐Module Microfluidics Enables Real‐Time Multiplexed Detection of Blood Insulin and Glucagon
Source: Adv Sci (Weinh). 2025 Apr 25;12(29):2412185. doi: 10.1002/advs.202412185 (PMC12362770; doi:10.1002/advs.202412185)
Supplement: Supplementary file 1 — Supporting Information [file ADVS-12-2412185-s002.docx]

Supporting Information for “A Bead-Based Quantum Dot Immunoassay Integrated with Multi-Module Microfluidics Enables Real-Time Multiplexed Detection of Blood Insulin and Glucagon”

Hesam Abouali*^,1,2^, Sanjana Srikant*^,1,2^, Md Fahim Al Fattah*^,1,2^, Nicole G. Barra^3^, Darryl Chan^3^, Dayan Ban^++,1,2^, Jonathan D. Schertzer^++,3,4,5^, Mahla Poudineh^++,1,2^

^1^ Department of Electrical and Computer Engineering, University of Waterloo, Waterloo, ON, N2L 3G1, Canada

^2^ Waterloo Institute for Nanotechnology, University of Waterloo, Waterloo, ON, N2L 3G1, Canada

^3^﻿ Department of Biochemistry and Biomedical Sciences, McMaster University, Hamilton, ON, L8S 4L8, Canada

^4^ Farncombe Family Digestive Health Research Institute, McMaster University, Hamilton, ON, L8S 4L8, Canada

^5^ Centre for Metabolism, Obesity and Diabetes Research, McMaster University, Hamilton, ON, L8S 4L8, Canada

*These authors contributed equally to the work.

++Correspondence to [mahla.poudineh@uwaterloo.ca](mailto:mahla.poudineh@uwaterloo.ca), [schertze@mcmaster.ca](mailto:schertze@mcmaster.ca), [dban@uwaterloo.ca](mailto:dban@uwaterloo.ca)

**Figure S1 – Insulin and glucagon BQI assay benchtop validation in buffer.**


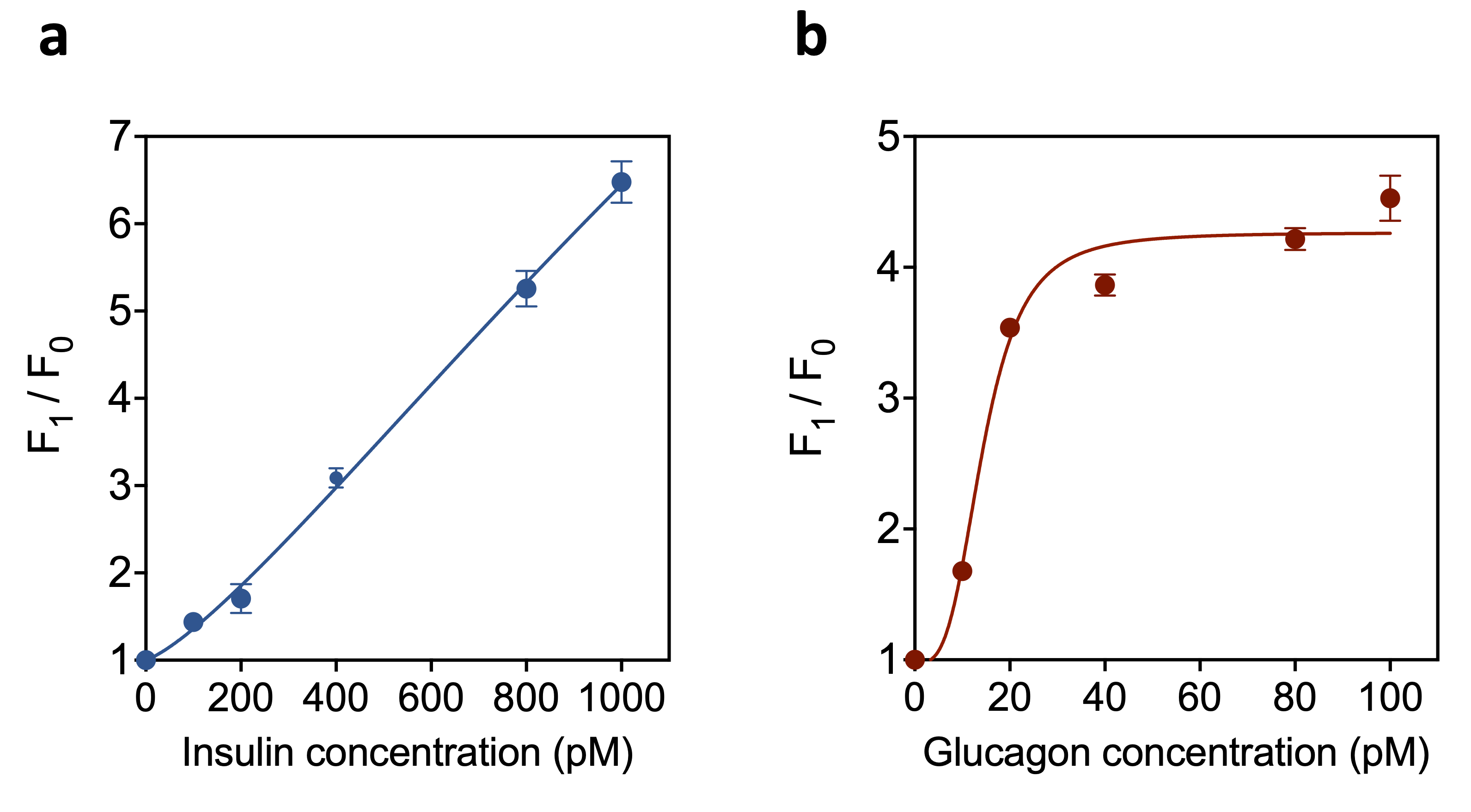


**Figure S1 – Benchtop BQIs in buffer.** a) shows the results for the insulin BQI in the buffer and b) shows the results for the glucagon BQI in the buffer. The purpose of these singular-analyte BQIs in the buffer was to confirm that the capture and detection antibodies are suitable for assay development prior to BQIs in whole blood. The data shows the mean ± standard deviation (SD) of three replicates. F_0_ is the fluorescent signal for the lowest concentration (0 pM), and F_1_ is the fluorescent signal for each sample.

**Figure S2 – Micromixer module design**


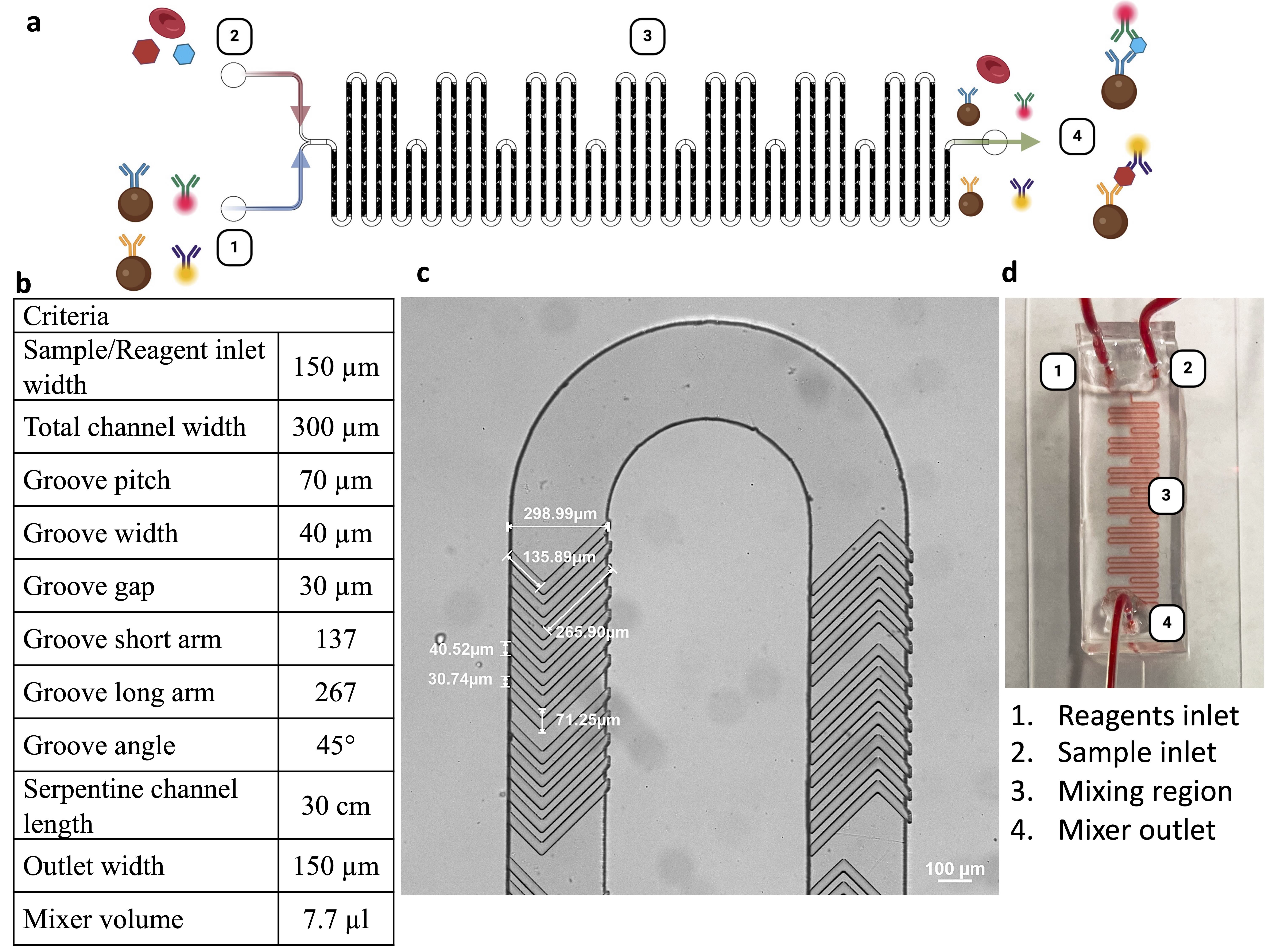


**Figure S2 – Micromixer module design.** The micromixer used in this device is a herringbone serpentine design which facilitates passive chaotic mixing within the microchannels. a) As shown in this panel, the design consists of two inlets that merge into a main channel. b) The table shows the dimensions of the micromixer and its geometrical features. c) Herringbone structures are fabricated on the top of the main channel with the dimensions shown in the microscopic images. d) The shows a fabricated micromixer with respective inlets and outlets. The solution shown in the figure within the device is food colorant for the purpose of microchannel visualization and does not demonstrate a blood flow stream within the device.

**Figure S3 - Glucagon assay specificity test.**

**Figure S3 – Glucagon assay specificity test*.*** Rat whole blood samples were spiked with GLP-1 (100 pM) or glucagon (10 pM). The spiked samples were evaluated by the QIRT-ELISA system for the specificity of the BQI for glucagon detection. The data shows the mean ± SD of three replicates. F_0_ is the average area under the peaks for the blank samples (0 pM), and F_1_ is the average area under the peaks for each sample with a higher concentration. The comparisons between groups are done with one-way ANOVA. p values: 0.1234 (ns), 0.0332 (*), 0.0021 (**), 0.0002 (***), <0.0001 (****).

**Figure S4 - Insulin BQI stability test.**

**Figure S4 – Insulin BQI stability test*.*** Rat whole blood samples were spiked with different concentrations of insulin and were evaluated by the QIRT-ELISA system for the stability of the BQI for insulin detection. On the first day, freshly made BQI reagents, including capture beads and the detection antibody-QDot conjugates, were used to conduct the on-chip BQI. After two weeks, the same previously made and used reagents were used for conduction of on-chip BQI for insulin detection. The data shows the mean ± SD of three replicates. F_0_ is the average area under the peaks for the blank samples (0 pM), and F_1_ is the average area under the peaks for each sample with a higher concentration. The comparisons between groups are done with one-way ANOVA. p values: 0.1234 (ns), 0.0332 (*), 0.0021 (**), 0.0002 (***), <0.0001 (****).

**Table S1 – The bead-based quantum dot-mediated immunoassay (BQI) kinetics model**

The duration required for thorough mixing relies on the diffusion coefficient (D) and the distance scale necessary for concentration homogenization, referred to as the striation length (st)^1^. This mixing time ($t_{mix}$) can be calculated as below:

$$t_{mix}= \frac{st^{2}}{D} (1)$$

Implementation of herringbone structures within the microfluidic mixer introduces chaotic flow streams in the microchannels. In this scenario, Baker’s transformation indicates a substantial decrease in striation length (st), showing an exponential decline, operating with the optimal flow rate^1^.

$st = st_{0} \times2^{-n}$ (2)

In this equation n is the number of chaotic advection structures, which is equal to 5 per half asymmetrical cycle, and in total 10 per full cycle in the mixer module of the QIRT-ELISA system. Thus, the mixing time in the herringbone microfluidic mixer can be calculated as:

$$t_{mix-chaotic}= \frac{s{t_{0}}^{2} \times2^{-10}}{D} = \frac{s{t_{0}}^{2}}{D\times2^{10}} = \frac{s{t_{0}}^{2}}{D_{eff}} \left( 3 \right)$$

Consequently, in the herringbone mixer, the diffusion is enhanced by about 1000 times.

As an example, we studied the kinetics of insulin assay. The binding kinetics of insulin antigen and its monoclonal capture antibody were measured previously by biolayer interferometry^2^. The association and dissociation values were measured experimentally as k_a_ = 2.8 x 10^4^ M^-1^s^-1^, and k_d_ = 0.0061 s^-1^, respectively. The diffusion-limited association rates of protein-protein complexes have also been reported to be in the 10^5^–10^6^ M^−1^s^−1^ range^3^.

The Smoluchowski results demonstrate that the diffusion-limited association rate constant (k_a_) can be approximated as linearly proportional to the diffusion^4^. Thus, as shown previously, the chaotic mixing can potentially increase the diffusion by 1000 times, hence the k_a_ value can potentially be in the range of 10^7^-10^9^ M^−1^s^−1^. However, the association rate, k_a_, cannot exceed the limit of 10^6^ M^-1^s^-1^ due to the orientational constraints of biomolecular interaction^3^. Thus, we assume in our model that it is equal to the upper bound of this range i.e., k_a_ = 10^6^ M^-1^s^-1^. Considering the measured value of k_d_ = 0.0061 s^-1^ ^2^, the equilibrium constant (K_D_) is K_D_ = k_d_/k_a_ = 6.1 $\times$ 10^-9^ M.

We follow the rate law for a protein-ligand reversible reaction^5^. This rate considers the concentrations of both reactants ([A]: [insulin] and [B] = [capture antibody]) and the following initial conditions.

$$\left[ A \right]+\left[ B \right]\rightleftharpoons\left[ AB \right]$$

$$\frac{d[AB]}{dt}= k_{a}\left[ A \right]\left[ B \right]-k_{d}\left[ AB \right] (4)$$

$$\left[ A \right]=\left[ A_{0} \right]-\left[ AB \right], \left[ A_{0} \right]\gg\left[ AB \right] at t=0$$

$$\left[ B \right]=\left[ B_{0} \right]-\left[ AB \right]$$

Assuming [A_0_] (initial concentration of insulin antigen) and [B_0_] (initial concentration of capture antibody on the bead surface) to be constant at t = 0, then we will have:

$$\frac{d[AB]}{dt}= k_{a}\left[ A_{0} \right]\left[ B_{0} \right]-(k_{a}\left[ A_{0} \right]+k_{d})\left[ AB \right] (5)$$

We assume [AB] as a conjugate with fluorescence signal F, then we get the solution of Equation 5 for F as follows^5^:

$$\frac{d[AB]}{(k_{a}\left[ A_{0} \right]\left[ B_{0} \right]-(k_{a}\left[ A_{0} \right]+k_{d})\left[ AB \right])}=dt (6)$$

$F\left( t \right) = exp(-\left( k_{a}\left[ \mathrm{insulin} \right]t+k_{d}t \right)$) + β (7)

$$at t=0:F_{min}=F_{background}= \alpha+\beta$$

$$at t=\infty:F_{max}= \beta$$

We will finally have the following equation:

$$F= F\left( t \right)- F_{background} = F_{max}\left( 1-\exp\left( -k_{a}\left[ insulin \right]t - k_{d}t \right) \right) (8)$$

$$\& F_{max}= \frac{A\times\left[ insulin \right]}{\left[ insulin \right]+ K_{D}} (8)$$

Where F_background_ is the background signal and A is an experimentally determined proportionality constant. We plotted this equation for a duration of 900 seconds with the respective parameter values for different concentrations of insulin (100 pM, 200 pM, 400 pM, and 1000 pM) (Figure 2c). We observe that it takes about 400 seconds for the complex to be in equilibrium. This plot is also shown for the initial 30-second time frame (Figure 2c).

A previous experimental study also quantitatively examines the binding kinetics of immunoglobulin G antigen to its fluorescently tagged detection antibody. The measured k_a_ = 3.8×10^5^ M^-1^s^-1^ and k_d_ = 3.15×10^-3^s^-1^ correlate well with the first section of our kinetics study^5^. Since the second reaction in the QIRT-ELISA system is happening within the micromixer as well, we choose the upper bound for k_a_, i.e. 10^6^ M^-1^s^-1^. We applied Equation 8 with the parameter values from the mentioned study for antigen-detection antibody formation. A similar trend is seen for the second step of the assay as well (Figure 2c). As shown in these figures, the output signal profile increases despite lower concentration of detection antibody.

**Table S1** – Parameters for the binding kinetics of the assay.

|  | **Capture Antibody – Antigen** | **Antigen – Detection Antibody** | **Unit** |
| --- | --- | --- | --- |
| k_a_ | 1.0 × 10^6^ | 1.0 × 10^6^ | M^−1^ s^−1^ |
| k_d_ | 6.1 × 10^-3^ | 3.15 × 10^-3^ | s^−1^ |
| K_D_ | 6.1 × 10^-9^ | 3.15 × 10^-9^ | M |

**Figure S5** –  **DLD module design**

Deterministic lateral displacement (DLD) microfluidic devices use specific arrangements of microposts to separate particles passively based on their size. Compared to active methods, which need external forces to be applied, this passive method is simple yet efficient. Within the microchannel, the microposts are positioned in a distinct geometric position. Design criteria such as size of microposts, the horizontal and vertical gap between the microposts, the number of micropost rows, and displacement angles between the rows define a criterion called the *critical diameter*. This critical diameter determines a cut-off value to separate particles based on their size (please see a comprehensive review^6^ of DLD design and principles). Particles larger than the critical diameter are displaced to another streamline at each successive micropost, since their hydrodynamic center is outside the width of the first streamline, while particles smaller than the critical diameter remain centered within the first streamline.

The DLD device of QIRT-ELISA was introduced in previous works^7,8^ and has a theoretical critical diameter of 14 µm. This means that particles smaller than 14 µm, which include red blood cells (RBCs), white blood cells (WBCs), platelets, and free-floating immunoassay reagents, will move in the first streamline. The immunoassay magnetic beads, which are larger than 14 µm and have a diameter of 15 µm, will be displaced as they traverse the length of the microfluidic device. Thus, a separation of assay beads and undesired components can be achieved. Figure S5 shows the details about the DLD device, including all design criteria and sections.


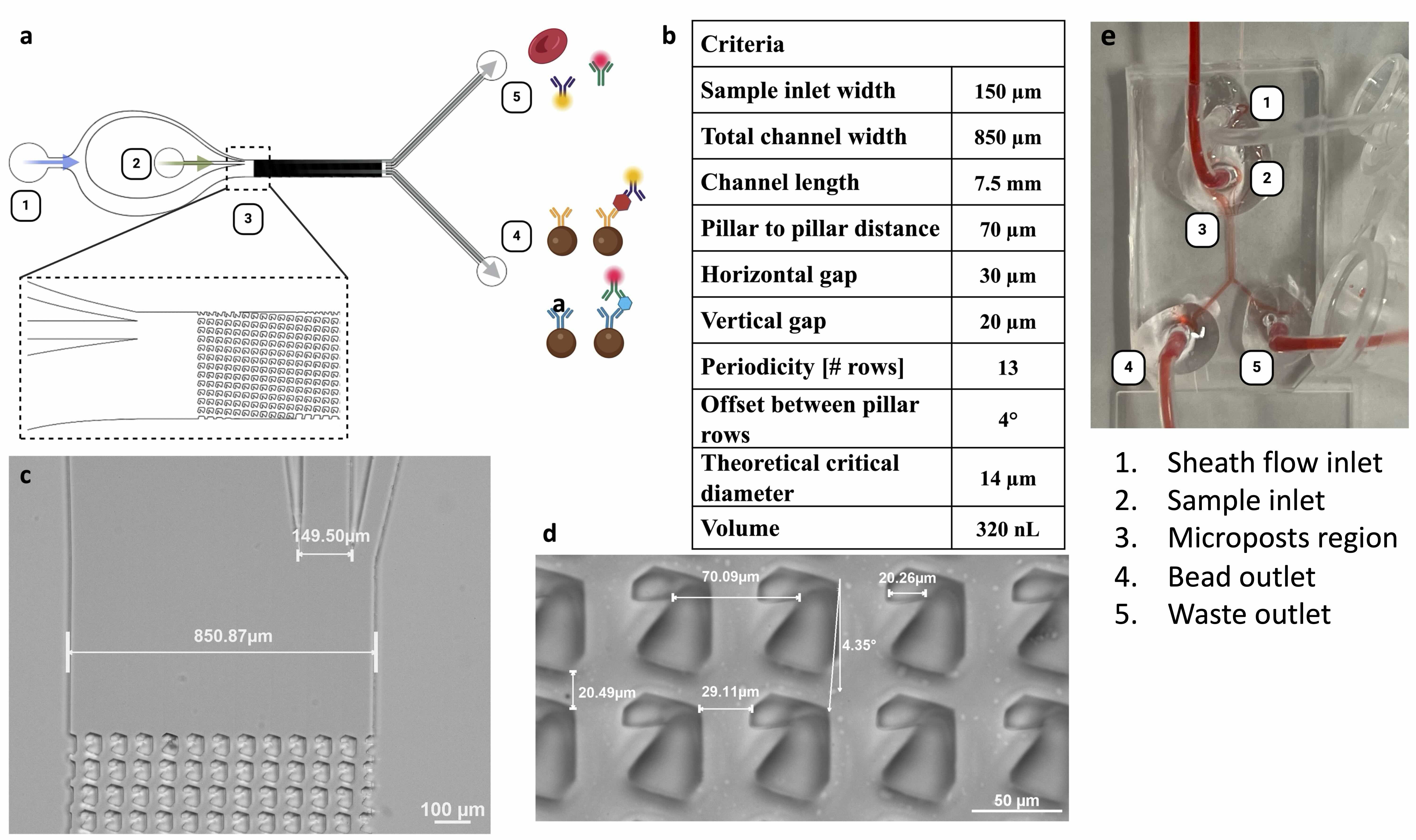


**Figure S5 – The washing module (DLD device).** a) shows the schematic of the DLD device with different parts labeled. A sheath flow enters the device (section #1) and helps the separation of the BQI beads from the undesired components entering the device (all from section #2). Formed BQI beads or unformed BQI beads move toward the bead outlet of the device (section #4) and unbound reagents including detection antibodies, antigens, and RBCs/WBCs are directed toward the waste outlet (section #5). The included table shows the design parameters for the utilized DLD device in the QIRT-ELISA system to achieve a critical diameter of 14 µm. b) the dimension for the fabricated DLD device is tabulated. c) shows a fabricated DLD device under the microscope with sample and sheath flow buffer inlets and the initial DLD arrays. d) shows a microscopic image of the DLD posts and related dimensions. e) a real image of the DLD device is shown as well. The solution shown in the figure within the device is food colorant for the purpose of microchannels visualization.

**Component evaluation in the DLD module**

The DLD module was evaluated for the status of all its components. There are four components within the DLD module: formed and unformed BQI beads, RBCs/WBCs, and unconjugated BQI reagents:

1. Formed bead-based quantum dot-mediated immunoassay (BQI): Shown in Figure 4A of the main manuscript and below, the fully integrated QIRT-ELISA system reaches a bead recovery rate of more than 80% and an outlet purity of 91%. The bead recovery rate means that more than 80% of the introduced beads in the reagents inlet of the first module (mixer) are exiting from the bead outlet of the DLD device, going into the detection module. This recovery rate is sufficient for the collection of data from the fully formed BQIs. The remaining 20% of the beads could be possibly directed to the waste outlet of the DLD device, or a fraction of them could remain inside the modules.
2. Unformed BQI: The unformed BQIs are also larger than the critical diameter of the DLD device and cannot be directed toward the waste outlet. These unformed BQIs are the result of an incomplete assay and have only the capture antibody on them. To explore this, the beads coated with the capture antibody were injected into the DLD module, and the number of beads was counted in both outlets. As shown in Figure S6, these beads followed the designed outlet and had a higher number in the bead outlet of the DLD module.


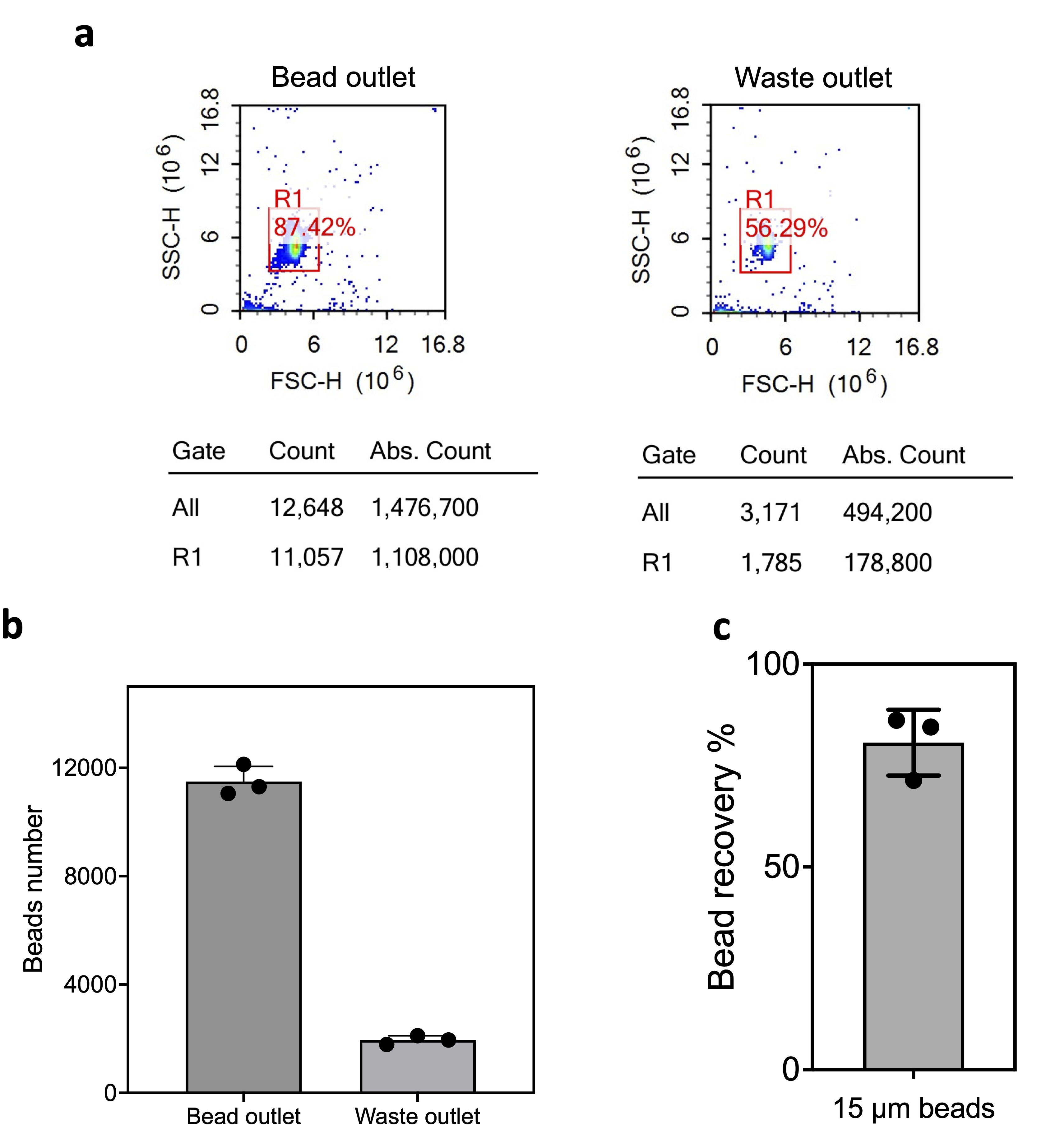


**Figure S6 – Control experiment for DLD output of the unbound BQI beads.** a) the flow cytometry results for a DLD device with the capture beads (12,000 beads in the inlet) in the absence of other components of the BQI reagents (antigen and QDot conjugated detection antibodies) suggest that capture beads follow the beads designated outlet, b) and c) the average recovery rate of beads is calculated at 77%. The data shows the mean ± SD of three replicates.

1. Unbound assay reagents: an experiment was conducted to demonstrate that the unconjugated reagents are directed toward the waste outlet of the DLD module. In this experiment, a free-floating detection antibody conjugated to Phycoerythrin organic dye (PE) was injected into a DLD device. This detection antibody follows the streamline to the waste outlet of the device. The outlets of the device were collected and analyzed for the spectra of the organic dye using a plate reader. As shown in Figure S7 and Video S1, the PE-conjugated detection antibody-PE moved in a zig-zag path toward the waste outlet since its size is smaller than the critical diameter (Figure S7).
2. RBCs/WBCs: The RBCs/WBCs are smaller than the critical diameter of DLD (= 14 µm) and are expected to be collected from the waste outlet. The outlet purity demonstrates that the waste outlet of the DLD device contains about 90% of the RBCs/WBCs which has entered the device from the whole blood samples (Figure S8).


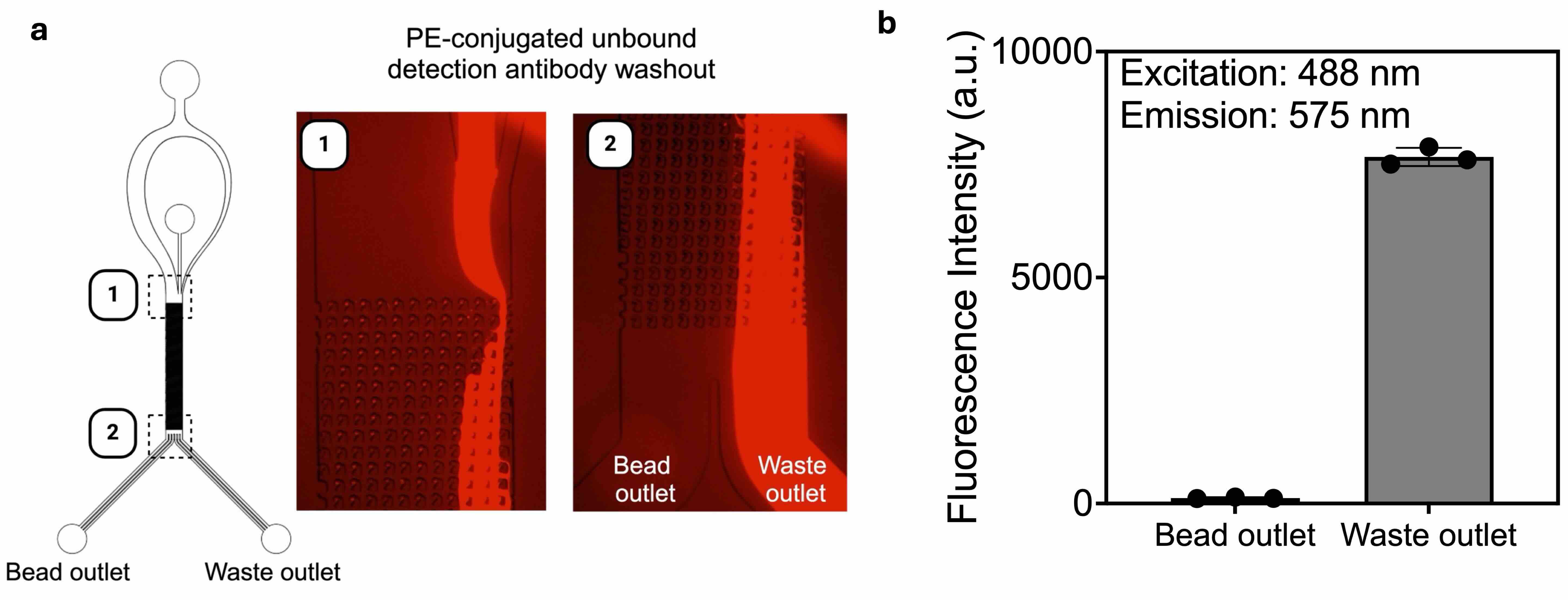


**Figure S7 – Control experiment for DLD module reagents wash characterization.** a) unbound detection antibody washout. Free-floating detection, which is conjugated to an organic dye, PE, was introduced into the DLD module. As the figure shows, the unbound detection antibody follows the waste outlet streamline as the size of the reagents is smaller than the critical diameter designed for the DLD module. b) the collected outlets were analyzed for the PE dye using a plate reader at its excitation peak (575 nm). The data shows the mean ± SD of three replicates.

**Figure S8 – Flow cytometry results for device configurations**

The performance of the modular and integrated devices regarding the bead recovery and purity were evaluated.

**Purity evaluation:** to evaluate the purity of the outlet from the modules or the fully integrated QIRT-ELISA device, the number of the RBCs/WBCs were counted in the waste and beads outlets and were compared with the help of SSC-FSC results from the flow cytometry with a proper gating for the cells (Figure S8, i and ii, gate P1). Then, the purity was calculated with the help of the following equation:

$$purity= \frac{number of RBCs and WBCs in waste outlet}{total number of RBCs and WBCs in both outlets}$$

**Bead recovery evaluation:** for bead the recovery calculations, the bead outlet of the device was collected and counted for the number of microbeads with the help of SSC-FSC results from the flow cytometry (Figure S8 iii, gate R1). This number was compared to the number of loaded microbeads into the device at the inlet.

$$recovery= \frac{number of the collected beads from. the bead outlet}{total number of the beads loaded into the device}$$

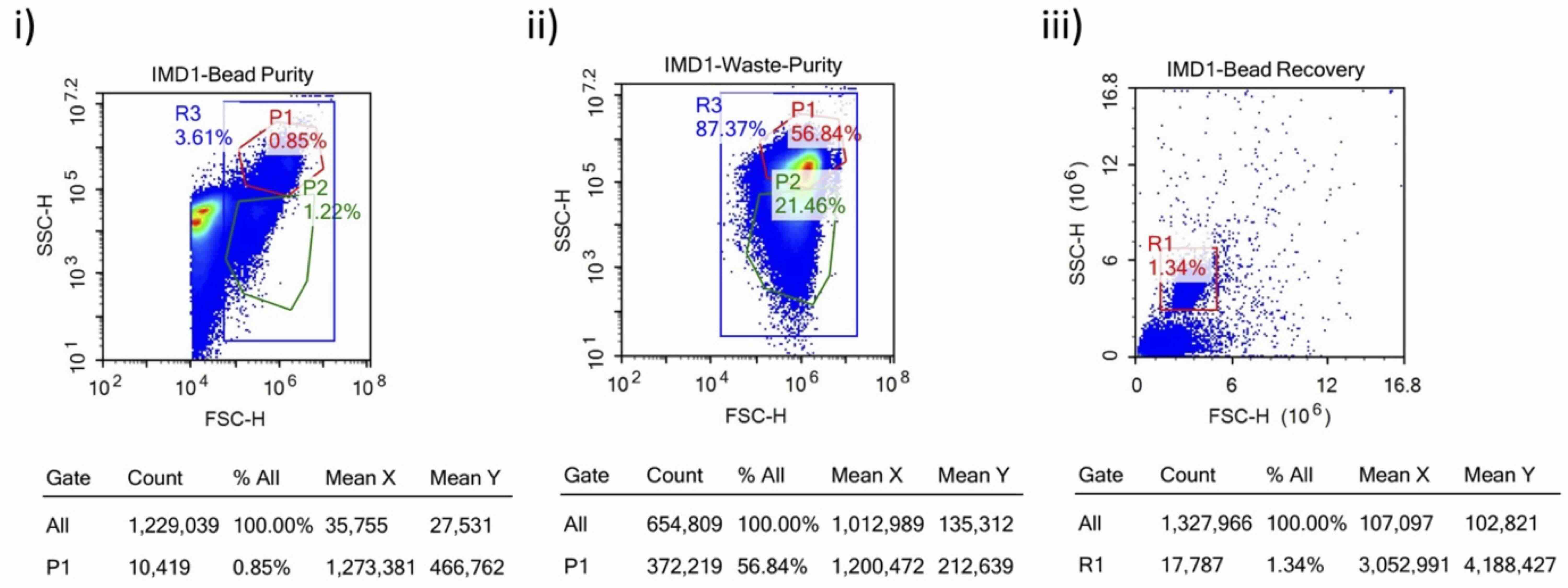


**Figure S8 – Evaluation of device purity and recovery. (**i) and (ii) show the results for the number of RBCs/WBC in the bead outlet and the waste outlet of the integrated QIRT-ELISA device. The graphs are gated for RBCs/WBCs (Gate P1), so the purity can be calculated. A higher number of RBCs/WBCs events can be seen in the waste outlet as expected (10,419 RBCs/WBCs in the bead outlet and 372,219 events in the waste outlet). (iii), shows the number of beads in the device’s bead outlet. This graph has been gated for 15 µm beads. (Gate R1) The number of events in this gate was compared to the number of beads loaded to the device within the reagents.

**Figure S9 – Detection device design**

**
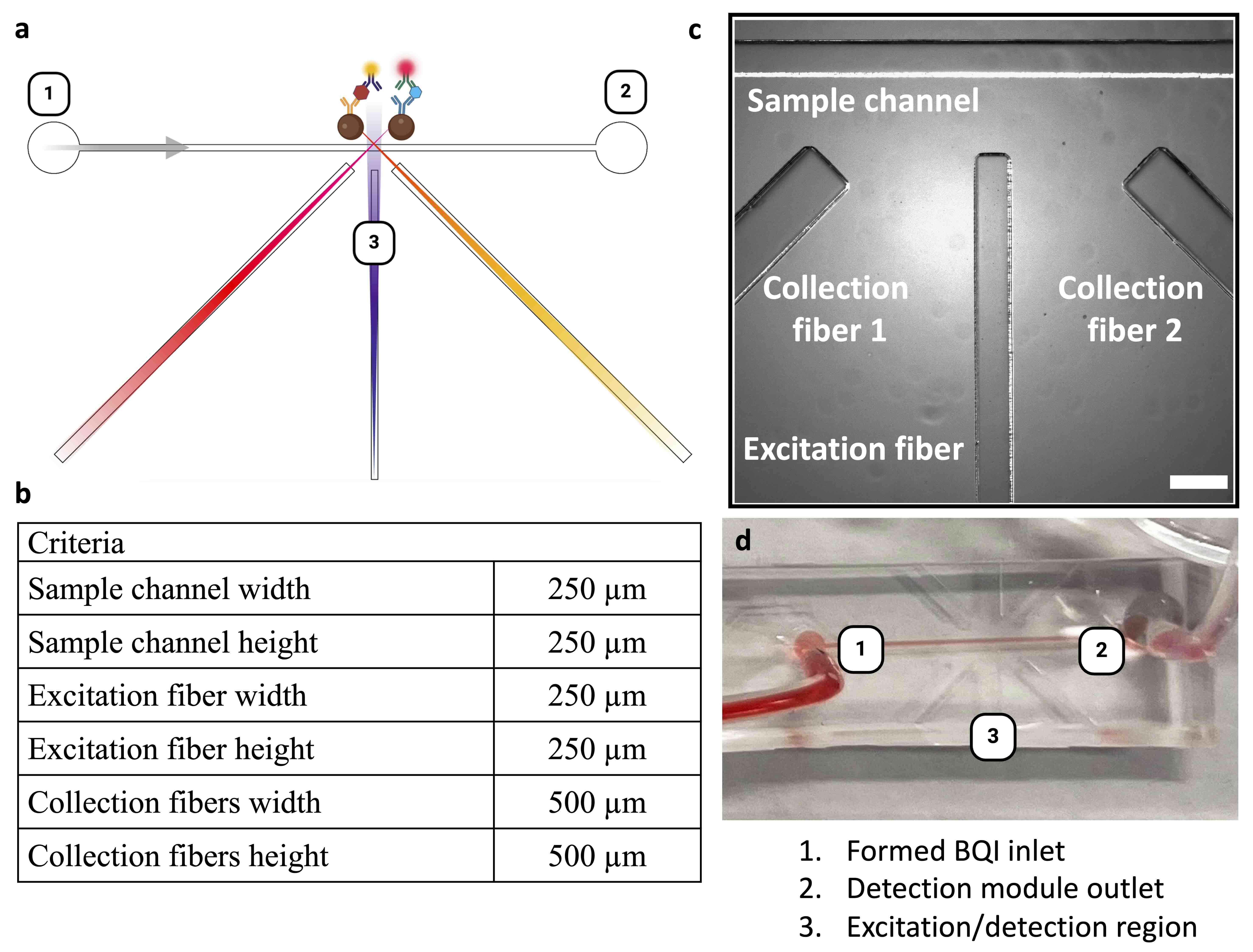
Figure S9 – Detection device design.** a) The detection device in this work includes a main channel and laser fiber grooves. The grooves house an excitation fiber (perpendicular) and two emission collection fibers (angled), to collect the data from the formed bead-based quantum dot-mediated immunoassay (BQI). b) The table shows the dimensions for the design of this module.

**Figure S10 – Optoelectronic Setup**


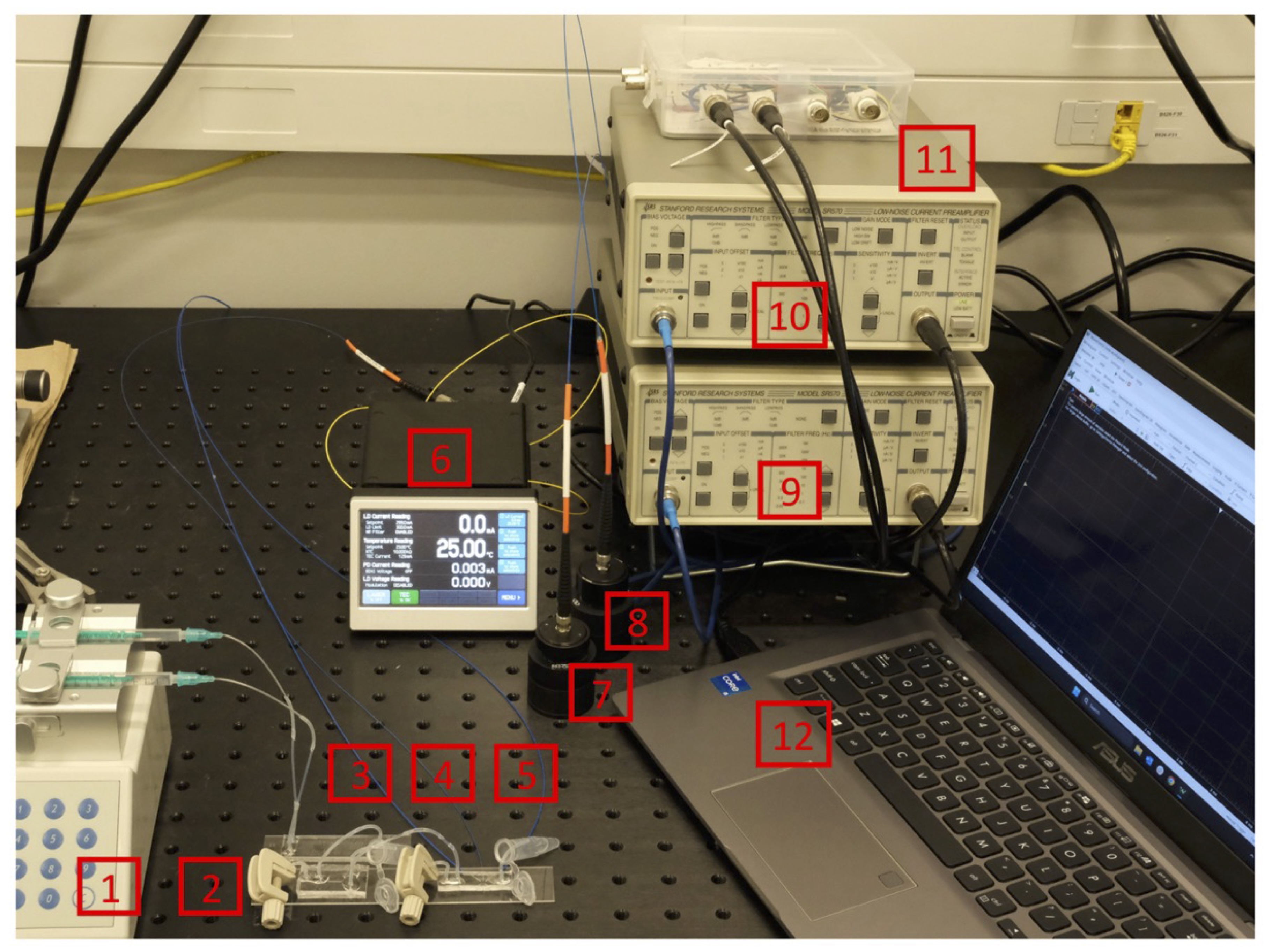


**Figure S10 – Optoelectronic setup for integration into the QIRT-ELISA system.** (1) A syringe pump is used to inject the sample and reagents into the device. (2) The integrated microfluidic device consisting of a micromixer, a DLD, and a detection module. Three optical fibers were inserted into the device where (3) is the first collection fiber (for QD605 nm), (4) is the excitation laser fiber, and (5) is the second collection fiber (for QD655 nm). (6) The excitation optical fiber is coupled with a 405 nm laser driver. (7) and (8) A photodetector and a band pass filter have been integrated for QDot 605nm and QDot 655 nm respectively. The fluorescence light goes through a band pass filter, passing the light and removing the background and scattered lights. The photodetectors capture the fluorescence light converting it to the equivalent electrical signal. (9) and (10) Two low noise current amplifiers amplify the current to a voltage, that is coupled with (11) an oscilloscope. (12) The signal outputs are recorded with a laptop.

**Figure S11 – The integrated QIRT-ELISA microfluidic device**


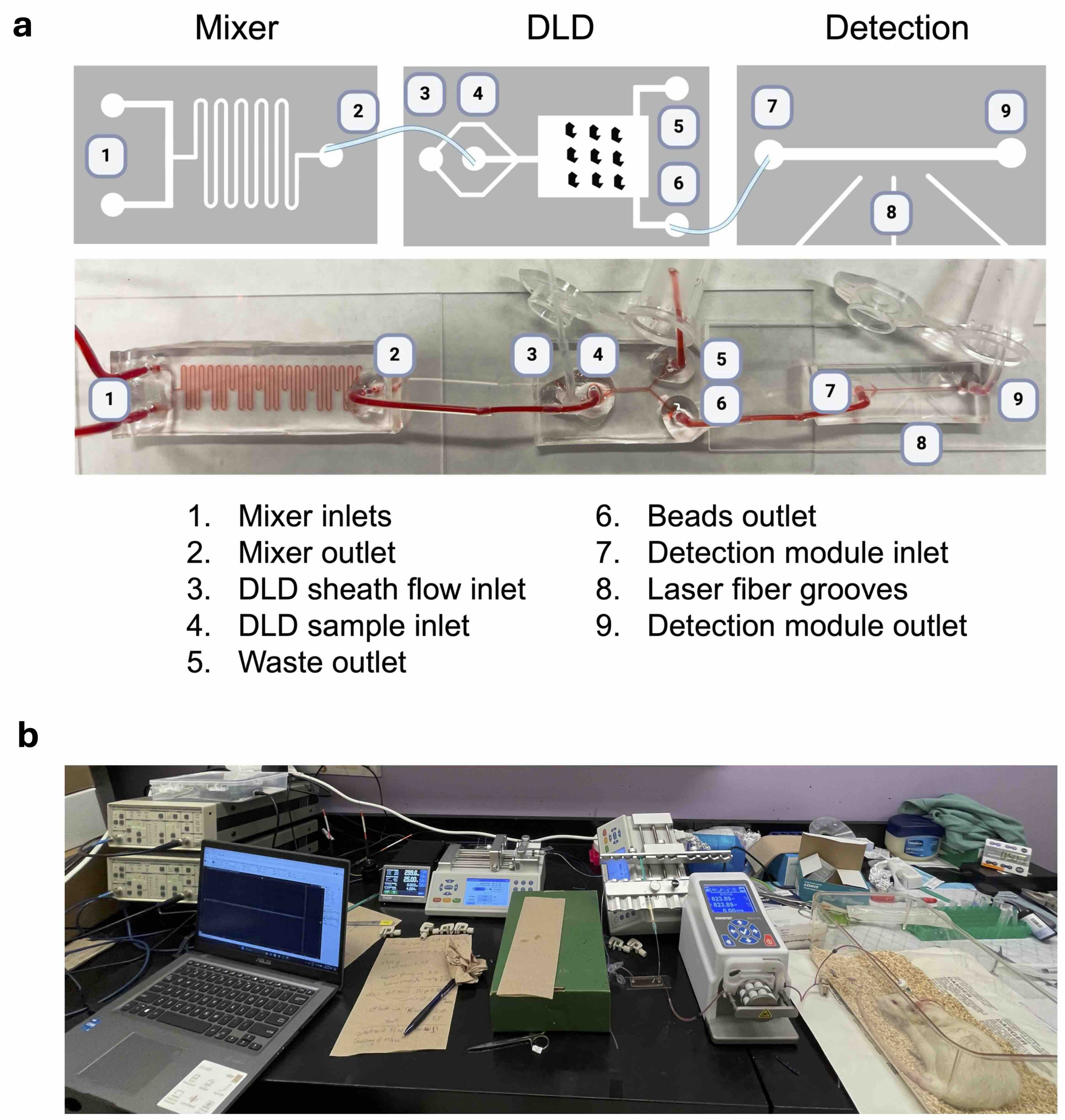


**Figure S11 – The integrated QIRT-ELISA system.** a) The individual modules were connected using silicon tubing. The outlet of the mixer, where BQI beads are formed, enters the sample inlet of the DLD module, and since the beads have a larger size than the critical diameter, they are separated from the undesired components (RBCs/WBCs, unconjugated reagents), and are directed into the detection module inlet. This module has the ability to integrate optoelectronic parts to measure fluorescence signals. An excitation fiber can be integrated into the perpendicular groove, and in the grooves with the 45-degree alignment at the same side of the excitation fiber, two collection fibers can be placed to collect the signals (not included in this image). The solution shown in the figure within the device is food colorant for the purpose of microchannels visualization, and does not demonstrate blood flow stream within device. b) The integrated QIRT-ELISA device was attached to a peristaltic pump which drew blood from a rat continuously. The withdrawn blood was also sampled at 0, 15, and 30-minute time points for conventional ELISA measurements from a valve incorporated into the pump tubing. The QIRT-ELISA system (under the covering green box to avoid interference of the room light on the fluorescence signals) measured insulin and glucagon levels simultaneously in a rat undergoing a GTT (a conscious rat in the shown experiment).

**Figure S12 – Measurement of insulin and glucagon with the integrated QIRT-ELISA system**


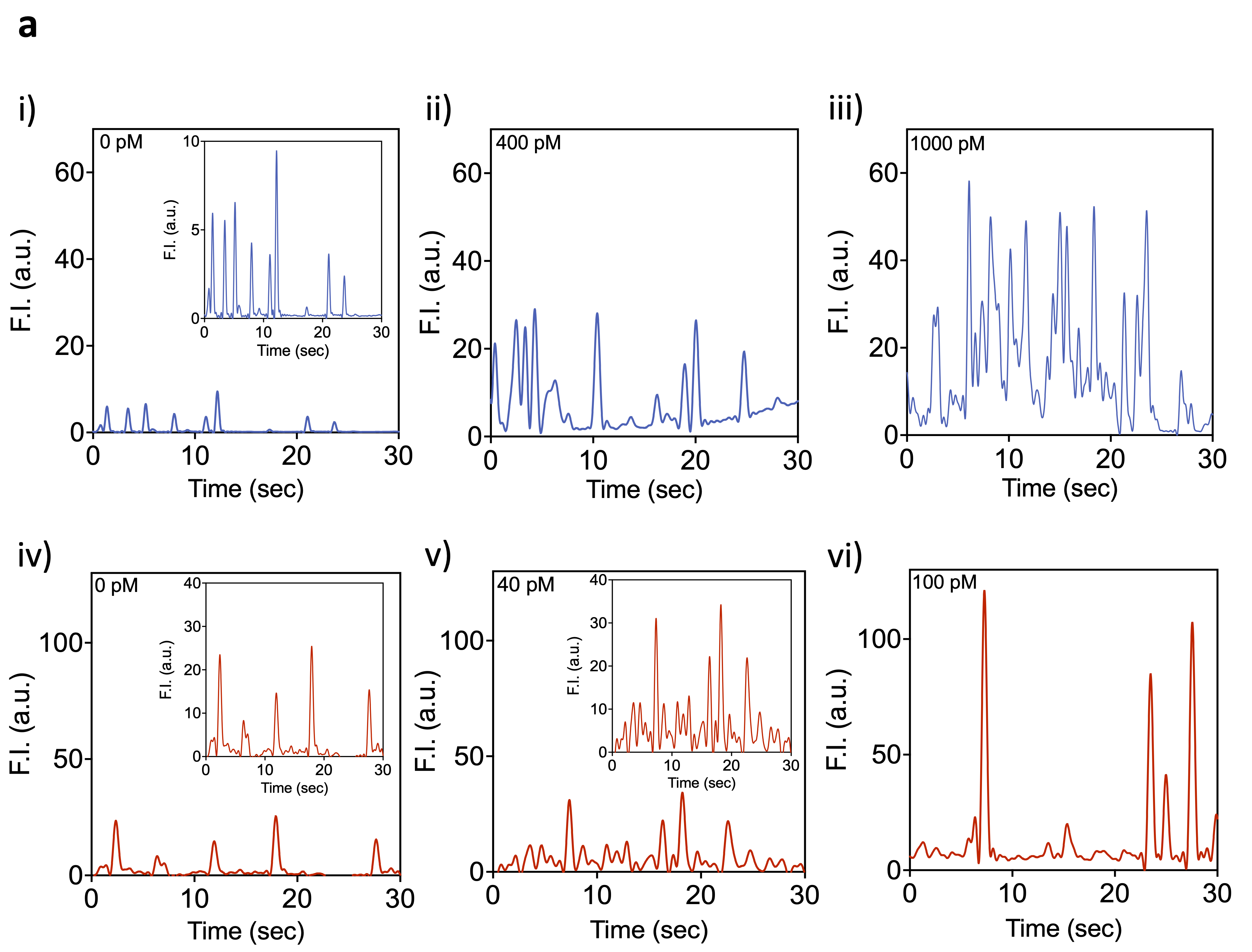


**Figure S12 – Integrated QIRT-ELISA device validation.** a) The fluorescence signals (F.I. (a.u.)) from on-chip BQIs. i, 0 pM, ii, 400 pM, iii, 1000 pM insulin signal scans. iv, 0 pM, v, 40 pM, vi, 100 pM glucagon signal scans. The scans were done for a duration of 60 seconds and the data are shown for a duration of 30 seconds in the graphs.

**Figure S13 – The validation of the developed app for peak area measurements.**


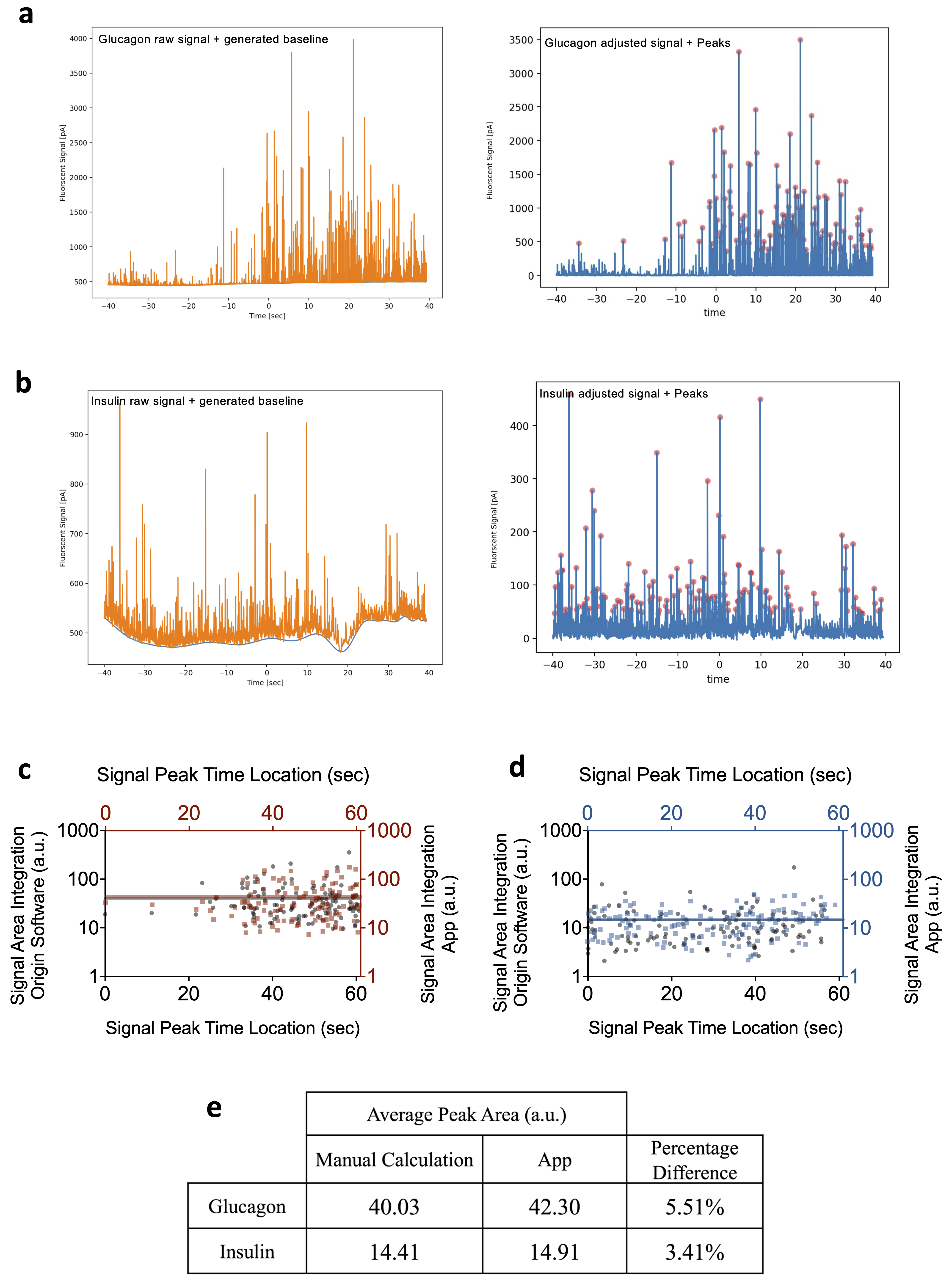


**Figure S13 – The validation of the developed app for peak area measurements.** A scan sample of multiplexed 100 pM insulin and 100 pM glucagon is shown here. The peak area integration was done with the developed app to facilitate quick measurements. The app was benchmarked against a manual data analysis using OriginPro 2023b software The raw data are first fit with a baseline (through iterative polynomial regression), and then the adjusted signals are analyzed for the peaks. A peak is defined if the signal exceeds 10% of the maximum peak amplitude. (a) shows the raw signal results and the generated baseline for glucagon in the left panel and the adjusted signal with the found peaks in the right panel. (b) The same data are shown for the insulin data in the same sample. The calculated peak areas by manual calculation (OriginPro software) and the developed app are compared for glucagon (c) and insulin (d). The results suggest that the average area peaks (black lines in the graphs for manual calculations, the red line for glucagon, and the blue line for insulin) are comparable, and have a very small difference in their values (e).

**Figure S14 – Discrete and continuous GTT experiment data correlation.** The collected data from both conventional ELISA and QIRT-ELISA are well-correlated (for samples which conventional ELISA had measurements). The insulin and glucagon concentrations from ELISA (in pM) and the normalized signal readouts (F_1_/F_0_ values) are tabulated.


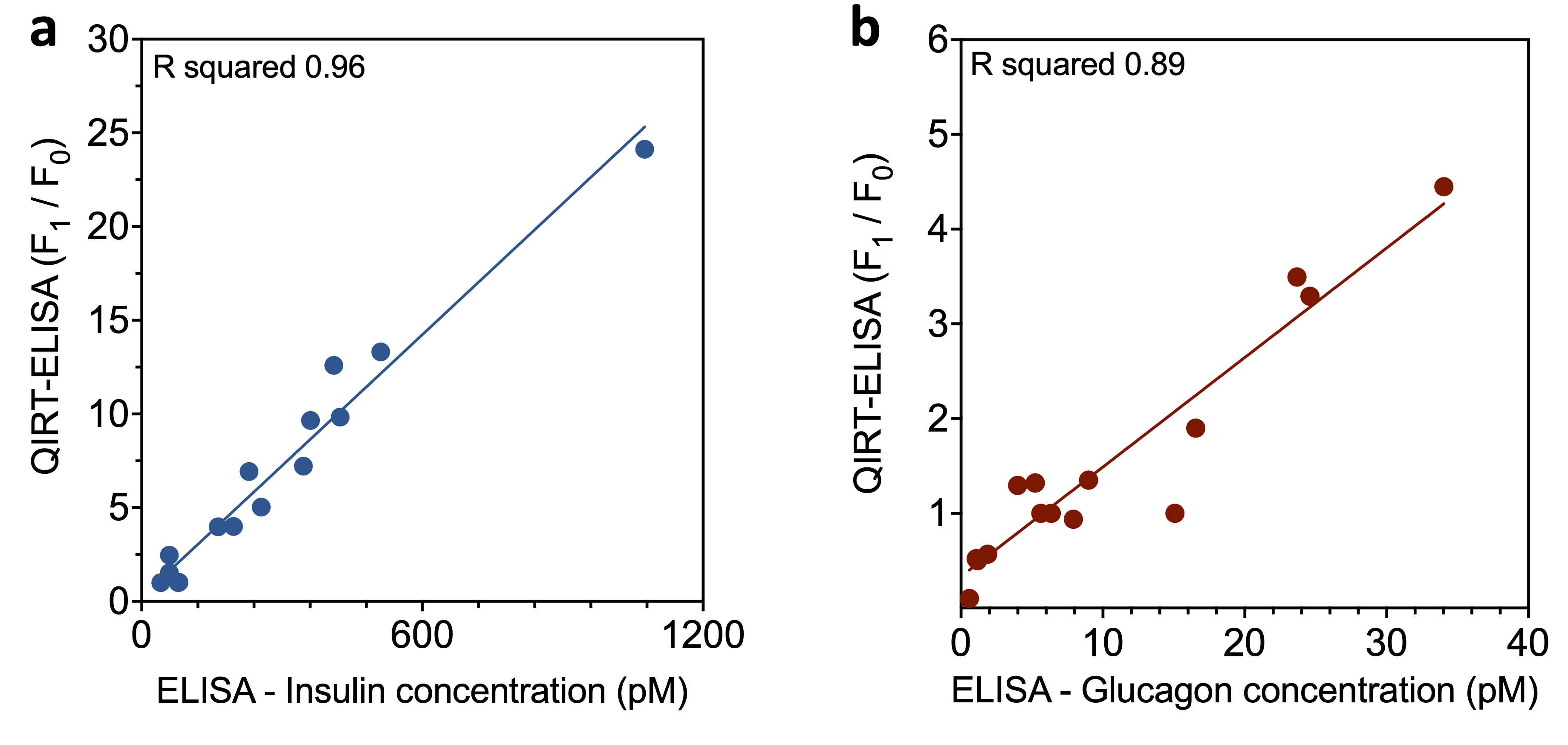


**Table S2 – GTT Experiment data.** Whole blood samples were analyzed for their insulin and glucagon levels by two methods at different time points. First, by conventional ELISA, and second by the QIRT-ELISA system. The concentrations from ELISA (in pM) and the normalized signal readouts (F_1_/F_0_ values) are tabulated in this table.

**
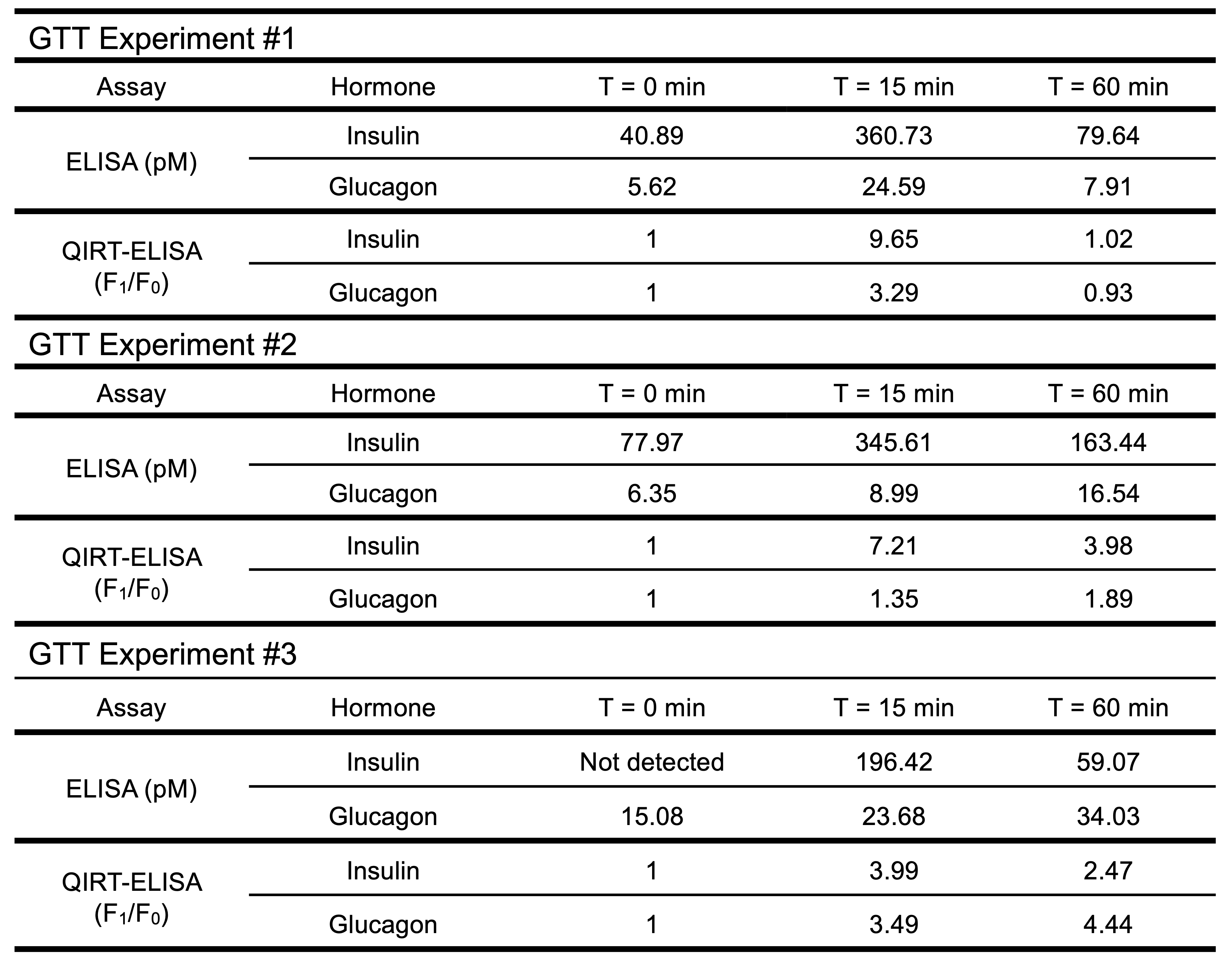
**

**Table S3 – Continuous GTT Experiment data.** Whole blood samples were analyzed for their insulin and glucagon levels by two methods at different time points by ELISA and continuously by the QIRT-ELISA platform. The concentrations from ELISA (in pM) and the normalized signal readouts (F_1_/F_0_ values) are tabulated.


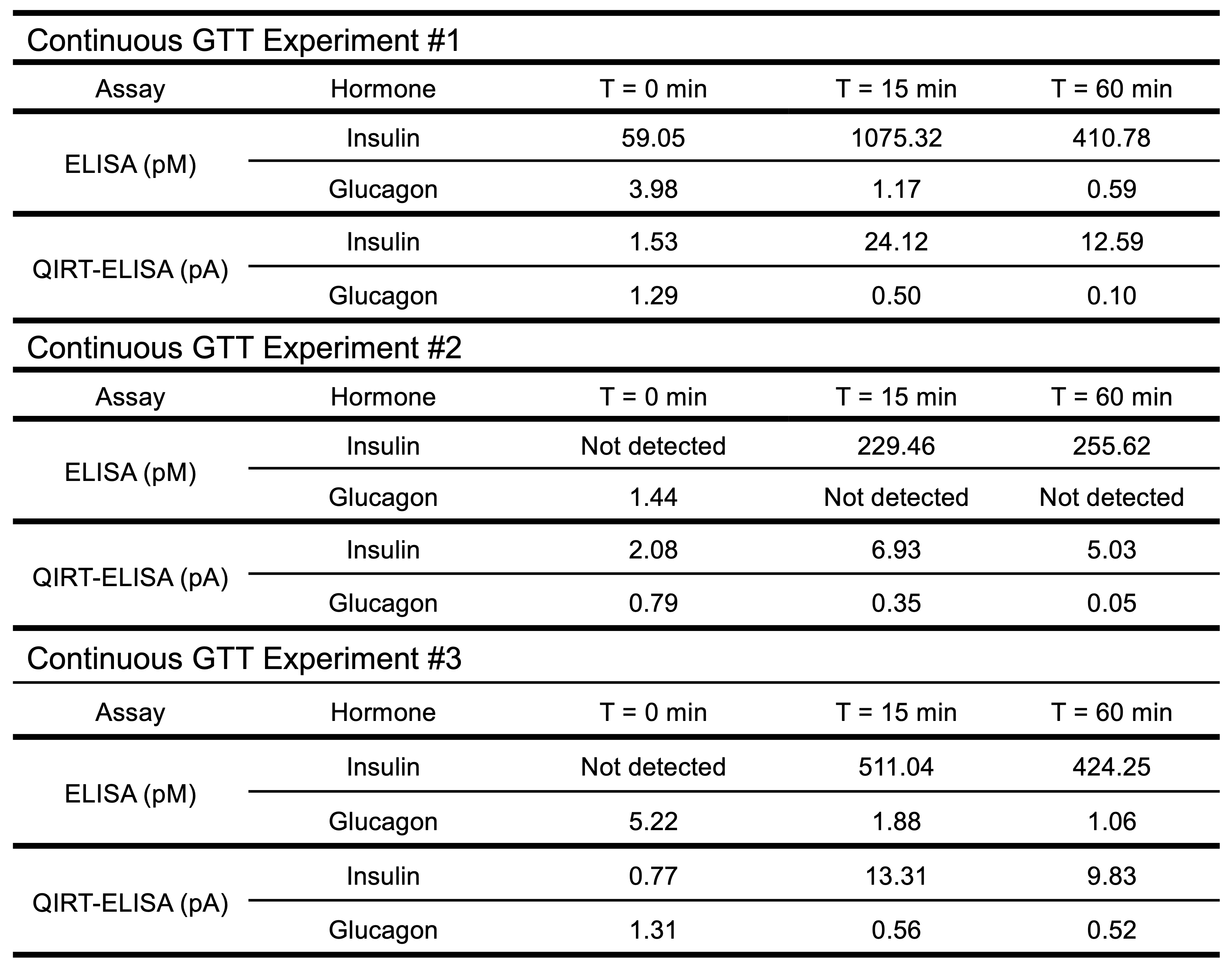


| Technology | Reagent Consumption | Preparation Time | Assay Time | Real-Time | Sensitivity | Limitation |
| --- | --- | --- | --- | --- | --- | --- |
| Conventional ELISA^9^ | High | Overnight | 6 hr | No | Low | Laborious, low sensitivity, scalability |
| nano-IMEX^10^ | Low | 18 hr | 40 min | No | High | Low throughput, complicated surface preparation |
| nano-HB^11^ | Low | 4.5 hr | 40 – 200 min | No | High | Low throughput, complicated surface preparation |
| MCR chip^12^ | Low | Overnight | 2 hr | No | High | Long assay time  No real-time measurement and multiplexing |
| RT-ELISA^2^ | Low | 4 hr | 1 min | Yes | High | Need for a bulky microscope.  Limitations in multiplexing |
| QIRT-ELISA  (This work) | Low | 3 hr | 1 min | Yes | High | - |

**Table S4 – ELISA Comparison.** Different microfluidic technologies have been developed to enhance the conventional ELISA for various applications. However, these systems still require further improvements.

**Table S5 - Information of reagents and buffers used in the current work.**

| Item | Vendor | ID |
| --- | --- | --- |
| carboxylate functionalized magnetic 15 µm microspheres | Spherotech | CM-150-10 |
| N-Hydroxysuccinimide | Sigma Aldrich | CAS # 6066-82-6 |
| 1-ethyl-3-(3-dimethylaminopropyl) carbodiimide | TCI America | CAS # 1892-57-5 25952-53-8 |
| 2-(N-morpholino) - ethanesulfonic acid | Sigma Aldrich | CAS # 4432-31-9 |
| SiteClick Antibody Labeling kit QDot 655 | Thermofisher | Catalog # S10453 |
| Qdot 605 Streptavidin Conjugate | Thermofisher | Catalog # Q10101MP |
| RBC Lysis Buffer | Invitrogen | Catalog # 00-4333-57 |
| Molecular Biology Grade Sucrose | Thermofisher | CAS # 57-50-1 |
| Phosphate-Buffered Saline 10X | Sigma Aldrich |  |
| Tween-20 | Sigma Aldrich | CAS # 9005-64-5 |
| Bovine Serum Albumin | Sigma Aldrich | CAS # 9048-46-8 |

**References**

1. Song, H., Bringer, M. R., Tice, J. D., Gerdts, C. J. & Ismagilov, R. F. Experimental test of scaling of mixing by chaotic advection in droplets moving through microfluidic channels. *Appl. Phys. Lett.* **83**, 4664–4666 (2003).

2. Poudineh, M. *et al.* A fluorescence sandwich immunoassay for the real-time continuous detection of glucose and insulin in live animals. *Nat. Biomed. Eng.* **5**, 53–63 (2021).

3. Schlosshauer, M. & Baker, D. Realistic protein–protein association rates from a simple diffusional model neglecting long-range interactions, free energy barriers, and landscape ruggedness. *Protein Sci.* **13**, 1660–1669 (2004).

4. Qin, S., Cai, L. & Zhou, H.-X. A method for computing association rate constants of atomistically represented proteins under macromolecular crowding. *Phys. Biol.* **9**, 66008 (2012).

5. Lin, C.-H. *et al.* Quantitative measurement of binding kinetics in sandwich assay using a fluorescence detection fiber-optic biosensor. *Anal. Biochem.* **385**, 224–228 (2009).

6. McGrath, J., Jimenez, M. & Bridle, H. Deterministic lateral displacement for particle separation: a review. *Lab Chip* **14**, 4139–4158 (2014).

7. Gomis, S. *et al.* Single-Cell Tumbling Enables High-Resolution Size Profiling of Retinal Stem Cells. *ACS Appl. Mater. Interfaces* **10**, 34811–34816 (2018).

8. Poudineh, M. *et al.* Continuous detection of glucose and insulin in live animals. *Nat. Biomed. Eng.* (2020) doi:10.1101/2020.01.22.916106.

9. Dudal, S. *et al.* Assay Formats: Recommendation for Best Practices and Harmonization from the Global Bioanalysis Consortium Harmonization Team. *AAPS J.* **16**, 194–205 (2014).

10. Zhang, P., He, M. & Zeng, Y. Ultrasensitive microfluidic analysis of circulating exosomes using a nanostructured graphene oxide/polydopamine coating. *Lab Chip* **16**, 3033–3042 (2016).

11. Zhang, P. *et al.* Ultrasensitive detection of circulating exosomes with a 3D-nanopatterned microfluidic chip. *Nat. Biomed. Eng.* **3**, 438–451 (2019).

12. Yafia, M. *et al.* Microfluidic chain reaction of structurally programmed capillary flow events. *Nature* **605**, 464–469 (2022).
